# The nuclear pore protein NUP98 impedes LTR-driven basal gene expression of HIV-1, viral propagation, and infectivity

**DOI:** 10.3389/fimmu.2024.1330738

**Published:** 2024-02-21

**Authors:** Kumaraswami Chintala, Sriram Yandrapally, Warisha Faiz, Chhaya Rani Kispotta, Satarupa Sarkar, Krishnaveni Mishra, Sharmistha Banerjee

**Affiliations:** Department of Biochemistry, School of Life Sciences, University of Hyderabad, Hyderabad, India

**Keywords:** nuclear pore complexes, NUP98, HIV-1 LTR, transcription, viral gene expression

## Abstract

Nucleoporins (NUPs) are cellular effectors of human immunodeficiency virus-1 (HIV-1) replication that support nucleocytoplasmic trafficking of viral components. However, these also non-canonically function as positive effectors, promoting proviral DNA integration into the host genome and viral gene transcription, or as negative effectors by associating with HIV-1 restriction factors, such as MX2, inhibiting the replication of HIV-1. Here, we investigated the regulatory role of NUP98 on HIV-1 as we observed a lowering of its endogenous levels upon HIV-1 infection in CD4^+^ T cells. Using complementary experiments in NUP98 overexpression and knockdown backgrounds, we deciphered that NUP98 negatively affected HIV-1 long terminal repeat (LTR) promoter activity and lowered released virus levels. The negative effect on promoter activity was independent of HIV-1 Tat, suggesting that NUP98 prevents the basal viral gene expression. ChIP-qPCR showed NUP98 to be associated with HIV-1 LTR, with the negative regulatory element (NRE) of HIV-1 LTR playing a dominant role in NUP98-mediated lowering of viral gene transcription. Truncated mutants of NUP98 showed that the attenuation of HIV-1 LTR-driven transcription is primarily contributed by its N-terminal region. Interestingly, the virus generated from the producer cells transiently expressing NUP98 showed lower infectivity, while the virus generated from NUP98 knockdown CD4^+^ T cells showed higher infectivity as assayed in TZM-bl cells, corroborating the anti-HIV-1 properties of NUP98. Collectively, we show a new non-canonical function of a nucleoporin adding to the list of moonlighting host factors regulating viral infections. Downregulation of NUP98 in a host cell upon HIV-1 infection supports the concept of evolutionary conflicts between viruses and host antiviral factors.

## Introduction

Human immunodeficiency virus-1 (HIV-1) is an etiological agent for acquired immunodeficiency syndrome (AIDS) in humans. The HIV-1 life cycle can be broadly divided into two stages: early and late events. Principally, the entry of the virus, reverse transcription of viral RNA, import of viral capsid into the nucleus, and integration of viral DNA into the host genome are considered as early stages, whereas post-integration steps such as transcription of viral genes, translation of viral proteins, and finally, release of viral particles are categorized as the late stages of HIV-1 replication. Equally essential as any other step during viral replication, viral gene transcription is an indispensable step for HIV-1 that depends on both viral and host transcription factors. The HIV-1 gene transcription occurs from the viral promoter 5' long terminal repeat (5'LTR) and is regulated by the viral regulatory protein Tat and a plethora of host transcription factors. Nearly 50 transcription factors are either predicted or shown to bind HIV-1 5'LTR and regulate the activity of this viral promoter (1). Yet, how these factors regulate viral gene expression and whether they act in a concerted manner are still open questions to investigate. Although many studies focused on identifying host transcription factors that regulate HIV-1 gene transcription to find a host-directed therapeutic target to cure HIV-1/AIDS, our understanding of molecular events underlying the HIV-1 gene expression regulation is still unfolding with newer players (2–4).

Nuclear pore complexes (NPCs) are cellular transport machineries embedded in the nuclear envelope that separates the cytosolic compartment from that of the nucleus in a typical human cell, providing an important basis for nucleocytoplasmic trafficking (5, 6). NPCs are built from nearly 30 different nucleoporins (NUPs), and these NUPs are arranged in multiple copies and occupy specific positions as subcomplexes in the NPC (5, 7, 8). As part of an NPC, NUPs regulate nucleocytoplasmic shuttling of molecules larger than 40 kDa between the nucleus and cytosol, conferring to the homeostasis of the cell. Apart from their well-known function in NPCs, many NUPs have been shown to have off-NPC functions, i.e., the regulation of gene expression, division of a cell, and maintenance of transcriptional memory (9–12). Consistent with these data, several microscopy studies indicate the presence of NUPs such as NUP153 and NUP98 in the nucleoplasm suggesting that NUPs are not only part of the static structures but also dynamically move on and off the NPCs and may play off-NPC roles under certain conditions (13, 14). Moreover, several studies show that the composition of NPCs changes both between and within the cells and that NUPs’ expression varies under certain conditions such as stimulation with interferons (IFNs) (15–21).

At several steps during its replication, HIV-1 hijacks NUPs to complete its life cycle in an infected cell. These steps include docking of the capsid (CA) onto NPC (NUP358), import of viral DNA (NUP153), integration of viral DNA into the host genome (NUP62, NUP98), and export of viral RNA (NUP98, NUP214, NUP62) (22–29). Furthermore, evidence shows that NUPs such as NUP358 and NUP153 are shown to be important for the HIV-1 integration site selection in the genome (26, 30, 31). Given their essential participation during HIV-1 replication, several proteomic studies have focused on and identified many NUPs that are dysregulated during HIV-1 infection (28, 32, 33). However, the functional association of many of the NUPs identified in these studies with respect to HIV-1 infection remains to be characterized. Kane et al. observed that both natural and manipulated changes in the composition of NUPs affected an HIV-1 infection and that the differential effect of NUP depletion on HIV-1 infection was dependent on cell type, cell cycle, and host factor cyclophilin A (CypA) (21). This study further demonstrates that HIV-1 utilizes several NUP-dependent pathways for viral entry into the nucleus. Accordingly, it was shown that HIV-1 utilizes a distinct set of NUPs for nuclear entry and requires CA and NUP interaction to do so (34). However, these studies could not exclude the possible participation of NUPs in other late steps of HIV-1 replication including transcription, and export of viral RNA. Yet, HIV-1–NUP interactions have also been implicated in myxovirus resistance 2 (MX2)-mediated HIV-1 restriction (21, 35). MX2, which is thought to prevent the importation of the viral preintegration complex, localizes to NPCs and interacts with many NUPs for the perturbation of HIV-1 infection (35–39). Thus, the critical understanding of both pro- and anti-HIV-1 roles for NUPs could be beneficial in finding novel anti-retroviral therapies.

Among NUPs, NUP98 is shown as one of the interferon-stimulated genes (ISGs) and is implicated in antiviral gene expression to limit viral infection in a *Drosophila* model (40). The gene for NUP98 expresses two alternatively spliced forms of mRNA: one encodes the shorter NUP98 protein and the other encodes the longer NUP98–NUP96 precursor protein. The longer NUP98–NUP96 precursor, upon autoproteolytic cleavage, gives rise to both full-length functional NUP98 (98 kDa) and NUP96 (96 kDa) proteins (41, 42). The N-terminal region of NUP98 is rich in the so-called GLFG (glycine-leucine-phenylalanine-glycine) motifs and is responsible for the interaction with protein factors such as RAE1, DHX9, and CBP/p300 (43–46). In the current study, we set out to understand the regulation of NUPs, which are implicated in the HIV-1 life cycle, during the late stages of the HIV-1 infection, i.e., the expression of the viral genes and the release of virions. We identified that NUP98 was downregulated under these conditions. While NUP98 was shown to promote the export of HIV-1 RNA via Rev-hCRM1-mediated transport from the nucleus to the cytosol across the nuclear membrane barrier (27), evidence also suggested that NUP98 was required for MX2-mediated HIV-1 restriction (35), pointing out multiple and conflicting roles for NUP98 as a pro- or anti-HIV-1 factor. We aimed to further understand the multifaceted function of NUP98 in HIV-1 infection and decipher the underlying mechanism driven by NUP98 that regulates HIV-1 propagation.

## Material and methods

All experiments were performed as per the guidelines of the Institutional Biosafety Committee.

### Cell lines and reagents

SupT1 cells (gifted by Dr. Shahid Jameel, International Centre for Genetic Engineering And Biotechnology, New Delhi, India) were grown in Roswell Park Memorial Institute’s medium (RPMI-1640, HiMedia, Cat. # AL162A) with 10% fetal bovine serum (FBS, Cat. #10270106, Invitrogen, USA), 100 μg/ml of streptomycin, and 100 U/ml of penicillin (Cat. # A001A, HiMedia, India). HEK293T cells (gifted by Dr. Reddy’s Institute of Life Sciences, Hyderabad, India) and TZM-bl cells (gifted by Prof. Ranga Udaykumar, Jawaharlal Nehru Centre for Advanced Scientific Research, Bengaluru, India) were grown in high glucose Dulbecco’s modified Eagle medium (DMEM, Cat. # AL066A, HiMedia, India) with 10% FBS, 100 μg/ml of streptomycin, and 100 U/ml of penicillin as recommended. Protein A/G agarose beads (Cat. # sc-2003) were purchased from Santa Cruz Biotechnology, USA. Antibodies against NUP155 (Cat. # ab199528), NUP133 (Cat. # ab155990), NUP107 (Cat. # ab73290), and HIV-1 p24 (Cat. # ab9071) were purchased from Abcam, USA. The antibody against NUP85 was purchased from Invitrogen, USA (Cat. # PA5-84522). Antibodies against NUP98 (Cat. # PAB196Hu01) and NUP62 (Cat. # PAC257Hu01) were purchased from Cloud-Clone Corp., USA. Antibodies against GAPDH (Cat. # sc-47724), GFP (Cat. # sc-9996), and HEXIM1 (Cat. # sc-390059) were purchased from Santa Cruz Biotechnology, USA. The antibody against β-tubulin (Cat. # AC008) was purchased from ABclonal, USA. The anti-NF-κB p65 antibody was purchased from Cell Signaling Technology, USA (Cat. # 4764T). The anti-HDAC1 antibody used in the study was a kind gift from Prof. Arunasree, University of Hyderabad, India (Cat. # BML-SA401-0100). Anti-rabbit HRP (Cat. # sc-2357) and anti-mouse HRP (Cat. # sc-516102) were purchased from Santa Cruz Biotechnology, USA. Lipofectamine 2000 (Cat. # 11668-019) was purchased from Invitrogen, USA.

### Plasmids

The plasmids including the HIV-1 molecular clone pNL4.3 and pHEF VSV-G were a kind gift from Dr. Udaykumar Ranga (Jawaharlal Nehru Centre for Advanced Scientific and Research, Bangalore, India) (47). The plasmids pLTR-Luc and pIndie-C1 were a kind gift from Dr. Debashis Mitra (National Centre for Cell Science, Pune, India) (48–52). The plasmids pEGFPC1, full-length GFP-NUP98, and GFP-NUP62 were a kind gift from Dr. Radha Chauhan (National Centre for Cell Science, Pune, India). The plasmids expressing different domains of NUP98 cloned in pEGFPC1 and full-length Myc-NUP98 cloned in pcDNA were a kind gift from Dr. Maureen Powers (Emory University, Atlanta, USA) (53). pSIV_AGM_-Luc-R^−^E^−^Δvif was a kind gift from Carsten Munk (Heinrich-Heine-University, Düsseldorf, Germany) (54). pNLC4.3GFP was a kind gift from Prof. Barbara Muller (University of Heidelberg, Germany) (55). The plasmid HIV-1 LTR-GFP was obtained from Addgene, USA (Cat. #115809). The pLKO plasmids expressing shRNA targeting NUP98 (TRCN0000291177) and scrambled shRNA were obtained from the ShRNA Resource Centre (Indian Institute of Science, Bangalore, India). HIV-1 Tat cloned in pcDNA3.1 was described previously (56). The deletion mutant constructs such as ΔNRE LTR, ΔNF-κB LTR, and ΔSP1 LTR were generated by site-directed mutagenesis using the respective primers listed in **Table 1** and the pLTR-Luc construct as a template.

**Table 1:** List of primers used in the study.

HIV-1 Env (RT-qPCR)	FP: 5'GCAGTGGGAATAGGAGCTTTGTTC3'
	RP: 5'GAGCTGTTGATCCTTTAGGTATCTTTCC3'
GAPDH (RT-qPCR)	FP: TGTTGCCATCAATGACCCCTT
	RP: CTCCACGACGTACTCAGCG
NUP98 (RT-qPCR)	FP: CCGTGATACCGAAGTTGAAAGC
	RP: AGATGCCTGCAAGACCTCAC
HIV-1 LTR (ChIP-qPCR) (+68 nt to +168 nt)	FP: 5'GCCTCAATAAAGCTTGCCTTGA3'
	RP: 5'TCCACACTGACTAAAAGGGTCTGA3'
ΔNRE LTR-Luc(SDM)	FP: 5'CTTACAAGGACCCTGAGAGAGAAGTGTTAG3'
	RP: 5'TCTCAGGGTCCTTGTAAGTCATTGGTCTTA3'
ΔNF-κB LTR-Luc (SDM)	FP: 5'TTGTTACAAAGGGAGGCGTGGCCTGGGCGG3'
	RP: 5'CGCCTCCCTTTGTAACAAGCTCGATGTCAA3'
ΔSp1 LTR-Luc (SDM)	FP: 5'ACTTTCCAGGAGCCCTCAGATGCTGCATAT3'
	RP: 5'TGAGGGCTCCTGGAAAGTCCCCAGCGGAAA3'
HIV-1 LTR (ChIP-qPCR) (NRE)	FP: 5'ATCTACCACACACAAGGCTACTTCC3'
	RP: 5'CCACTCTAACACTTCTCTCTCAGGGT3'
HIV-1 LTR (ChIP-qPCR) (NF-κB/Sp1)	FP: 5'TTTGACAGCCGCCTAGCATTTC3'
	RP: 5'CATCTGAGGGCTCGCCACTCC3'

### Transfections, virus preparations, and infections

In all the experiments unless otherwise stated, HEK293T cells were seeded overnight before transfection at 4 * 10^5^ cells per well in a six-well plate and then transfected or co-transfected with the indicated plasmids for 6 hours (h) at 37°C with 5% CO_2_ using Lipofectamine 2000. Cells were then added with fresh DMEM containing 10% FBS and further incubated for 48 h at 37°C with 5% CO_2_. For VSV-G pseudotyped HIV-1 NL4.3 virus preparation, HEK293T cells were seeded at 80% confluence before transfection in a six-well plate, and transfection was performed using the calcium phosphate method. Briefly, transfection mixtures were made with the plasmids pNL4.3 and pHEF VSV-G at 3:1 (total 3 μg per well) and sprinkled on the cells with DMEM containing 10% FBS, 100 μg/ml of streptomycin, and 100 U/ml of penicillin. Cells were then allowed for transfection for 6 h at 37°C with 5% CO_2_. The transfection medium was removed and fresh DMEM with 10% FBS, 100 μg/ml of streptomycin, and 100 U of penicillin was added to the cells, and cells were incubated at 37°C with 5% CO_2_ for the formation of HIV-1 NL4.3. Forty-eight hours post-transfection, the culture supernatant containing the virus was collected, centrifuged at 500*g* for 10 min for the removal of cell debris, and filtered through a 0.45-μm syringe-driven filter. The virus was then precipitated with 8.5% PEG and 0.3 M of NaCl at 4°C overnight. The virus was pelleted at 7,000*g* for 10 min at 4°C, resuspended in incomplete DMEM, and stored at −80°C. The concentration of viral p24 was estimated by p24 ELISA according to the manufacturer’s protocol (ABL, Cat. # 5447). SupT1 cells were infected by spinoculation, wherein cells were added with the required amount of the HIV-1 NL4.3 virus (2 ng of p24 equivalents/1 * 10^5^ cells) in complete RPMI medium with DEAE dextran (10 μg/ml), allowed for centrifugation at 350*g* for 40 min at 15°C, and incubated for 2 h at 37°C, 5% CO_2_ for viral entry. Then, cells were washed with PBS twice before they were incubated in a complete RPMI medium at 37°C with 5% CO_2_ for 4 days. HEK293T cells were incubated with the HIV-1 NL4.3 virus (2 ng of p24 equivalents/1 * 10^5^ cells) in a complete DMEM medium with DEAE dextran (10 μg/ml) for 4 h at 37°C, 5% CO_2_ for viral entry. Then, cells were washed with PBS twice before they were incubated in a complete DMEM medium at 37°C with 5% CO_2_ for 4 days.

### Lentivirus production and shRNA knockdown in HEK293T and SupT1 cells

For the knockdown of NUP98 in HEK293T cells, cells were seeded overnight before transfection at 4 * 10^5^ cells per well in a six-well plate and then transfected with shRNA-expressing plasmids for 6 h at 37°C with 5% CO_2_ using Lipofectamine 2000. Cells were added with fresh DMEM containing 10% FBS and further incubated for 48 h at 37°C with 5% CO_2_. Forty-eight hours post-transfection, cells were washed once with PBS and lysed in NP-40 buffer. The efficiency of the knockdown of NUP98 by shRNA was determined by Western blotting using anti-NUP98 antibody. The lentivirus used for the knockdown of NUP98 in SupT1 cells was prepared by transfecting HEK293T cells with packaging and transfer vectors as previously described (54). Briefly, HEK293T cells in a six-well plate were co-transfected with VSV-G encoding pMD2.G (250 ng), Gag-Pol encoding psPAX2 (1,000 ng), and pLKO.1-Puro (1,000 ng) harboring NUP98-specific shRNA sequences by the calcium phosphate method. Forty-eight hours post-transfection, the virus was collected, centrifuged at 500*g* for 10 min for the removal of cell debris, filtered through a 0.45-μm syringe-driven filter, and stored at −80°C. The infected SupT1 cells were transduced with the lentivirus containing either Sc shRNA or NUP98-specific shRNA sequences for 24 h. Seventy-two hours post-transduction, cells were harvested and analyzed for the depletion of NUP98 by Western blotting using the anti-NUP98 antibody.

### Western blotting

At indicated time points, cells were harvested, washed twice with PBS, and resuspended in NP-40 lysis buffer (50 mM of Tris pH 8.0, 150 mM of NaCl, 1.0% NP-40) with 1X protease inhibitor cocktail (PIC). Then, cells were vortexed for 30 min at 4°C, followed by centrifugation at 12,000 rpm for 20 min at 4°C. Protein lysates were collected and proteins were resolved on SDS-PAGE and transferred onto the nitrocellulose membrane. Membrane blots were incubated with primary antibodies diluted in blocking buffer (TBST with 1% BSA) at 4°C overnight on the shaker. All the primary antibodies used in the study were diluted at 1:2,000 in a blocking buffer. After three washes with TBST, blots were incubated with secondary antibodies conjugated to HRP at room temperature for 1 h on the shaker. The secondary antibodies such as the anti-rabbit HRP and anti-mouse HRP were diluted at 1:10,000 in blocking buffer. After three washes with TBST (Tris-buffered saline Tween 20, 0.1%), blots were developed using the chemiluminescence detection kit (Cat. # K-12045-D10, Advansta, USA). Using ImageJ-win64 software, the protein bands of interest were quantified and the values were normalized to that of the corresponding loading controls (GAPDH or tubulin) for each blot individually.

### RT-qPCR

At indicated time points, cells were harvested, washed twice with PBS, and resuspended in TRIzol. Total RNA was isolated and treated with DNase I to remove the contaminating genomic DNA. One microgram of RNA was used to obtain cDNA using the iScript cDNA synthesis kit (Cat. # 1708891, Bio-Rad, USA), which was then used as a template for the amplification of HIV-1 Env mRNA by iTaq Universal SYBR Green Supermix (Cat. # 172-5121, Bio-Rad, USA) using the primers listed in **Table 1**. The expression of HIV-1 Env mRNA was normalized to GAPDH mRNA as an internal control.

### Cell viability by trypan blue dye exclusion assay

The viability of the cells was determined by trypan blue dye exclusion assay as previously described (57). Briefly, cells were harvested at indicated time points and resuspended in PBS. Then, cells were mixed with equal volumes of trypan blue (0.4%), and both viable and non-viable cells were counted based on the dye exclusion in the hemocytometer. The percentage of viable cells was determined by dividing the viable cells by the total number of cells.

### Luciferase activity assay

HEK293T cells were seeded overnight before transfection at 1 * 10^5^ cells per well in a 24-well plate and then co-transfected with reporter plasmids along with the expression plasmids or molecular clone pNL4.3 for 6 h at 37°C with 5% CO_2_ using Lipofectamine 2000 (Invitrogen, USA). Cells then were added with fresh DMEM containing 10% FBS and further incubated for 48 h at 37°C with 5% CO_2_. Forty-eight hours post-transfection, cells were washed once with PBS and 120 μl of reporter lysis buffer (Cat. # E397A) was added to each well, followed by two freeze–thaw cycles at −80°C for cell lysis. Luciferase activity (RLU) in the cell lysates was measured using Luciferase assay reagent (Cat. # E1483) by Luminometer (Turner BioSystems, USA). For each sample, RLUs were normalized to the protein quantified by the BCA method according to the manufacturer’s protocol (Cat. # 786-570).

### TZM-bl reporter assay

Overnight before infection with HIV-1 NL4.3, TZM-bl cells were seeded in a 24-well plate at 1 * 10^5^ cells per well in DMEM medium with 10% FBS. Cells were then allowed for binding with HIV-1 NL4.3 (5 ng p24/well) for 3 h at 37°C with 5% CO_2_. Forty-eight hours post-infection, cells were washed once with PBS and 120 μl of reporter lysis buffer was added to each well, followed by two freeze–thaw cycles at −80°C for cell lysis. Luciferase activity (RLU) in the cell lysates was measured as mentioned above.

### Chromatin immunoprecipitation

HEK293T cells, 48 h post-co-transfection with Myc-NUP98 and LTR constructs, harvested from a six-well plate were washed with PBS and subjected to cross-linking with 1% formaldehyde for 15 min at 37°C, followed by quenching with 125 mM of glycine for 5 min at 37°C. Cross-linked cells were washed twice with PBS and sonicated in 200 μl of NP-40 lysis buffer for six cycles with pulses 20 s on and 30 s off on ice with 30% power. The supernatant containing chromatin fragments of approximately 400–800 bp was obtained by centrifugation at 12,000 rpm and 4°C for 20 min. The supernatants were incubated with protein A/G agarose beads preconjugated to anti-Myc antibody at 4°C overnight. For preconjugation, 2 μg of anti-Myc antibody was used for each sample. The immunoprecipitates with protein A/G agarose beads were pelleted down by centrifugation at 2,000 rpm for 2 min and washed with TBST. The antibody-protein-bound DNA was eluted after reverse cross-linking. The column-purified DNA quantified by spectrometry was used as a template for qPCR using the primers (**Table 1**) designed in the HIV-1 LTR promoter.

### Co-immunoprecipitation

HEK293T cells were transfected with either pcDNA or Myc-NUP98. Forty-eight hours post-transfection, cells were washed with PBS and lysed in NP40 buffer. Similarly, HEK293T cells infected with HIV-1 NL4.3 were transfected with either pcDNA or Myc-NUP98. Forty-eight hours post-transfection, cells were washed with PBS and lysed in NP-40 buffer. Protein A/G agarose beads were washed with TBST buffer and incubated with cell lysate for 4 h at 4°C to remove proteins that may non-specifically bind to the protein A/G agarose beads (preclearing step). After preclearing wherein the beads were removed by centrifugation at 2,000 rpm for 2 min, the supernatant was added to the fresh protein A/G agarose beads already conjugated with 2 μg of anti-Myc antibody and incubated overnight at 4°C on a rocker. After overnight incubation, beads were washed three times by centrifugation at 2,000 rpm for 2 min with TBST buffer. The samples with the beads were then dissolved in an SDS loading buffer and processed for Western blot analysis.

### Data and statistical analysis

All the experiments were performed at least three times. The represented values were the mean with standard deviation. For statistical analysis, Student’s paired *t*-test was conducted for all the experiments except otherwise mentioned, for which one-way ANOVA with Tukey’s multiple comparisons test was conducted using GraphPad Prism 5. P < 0.05, *P* < 0.01, and *P* < 0.001 were considered statistically significant and represented as *, **, and **, respectively. P>0.05 was considered as non-significant (NS).

## Results

### HIV-1 infection downregulates nucleoporin NUP98

**Figure 1:**
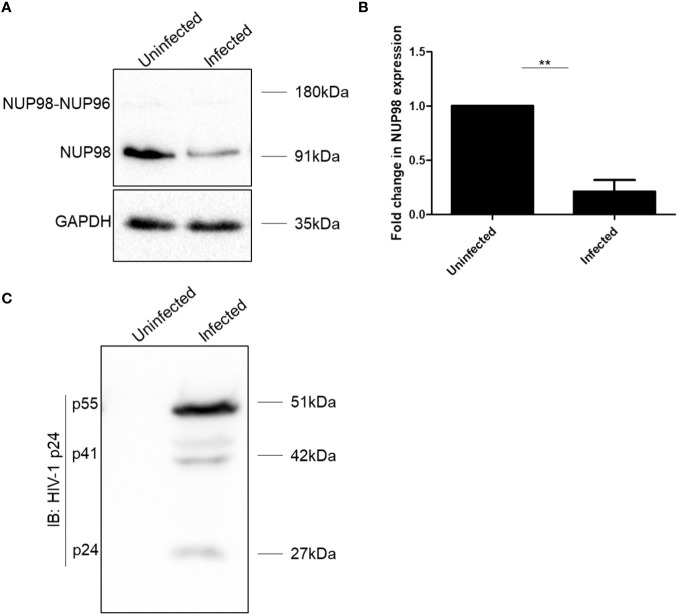
HIV-1 downregulates NUP98 in CD4^+^ SupT1 cells. **(A)** Representative Western blot showing endogenous NUP98 levels upon HIV-1 NL4.3 infection of SupT1 cells. Blots were probed with anti-NUP98 and anti-GAPDH antibodies. **(B)** Bar graphs representing the mean fold change of NUP98 expression relative to the uninfected cells. **(C)** Representative Western blot showing intracellular p55 levels upon HIV-1 NL4.3 infection of SupT1 cells. Blots were probed with anti-HIV-1 p24 antibodies. The experiments were performed at least three times. **, *P* < 0.01.

**Figure 2:**
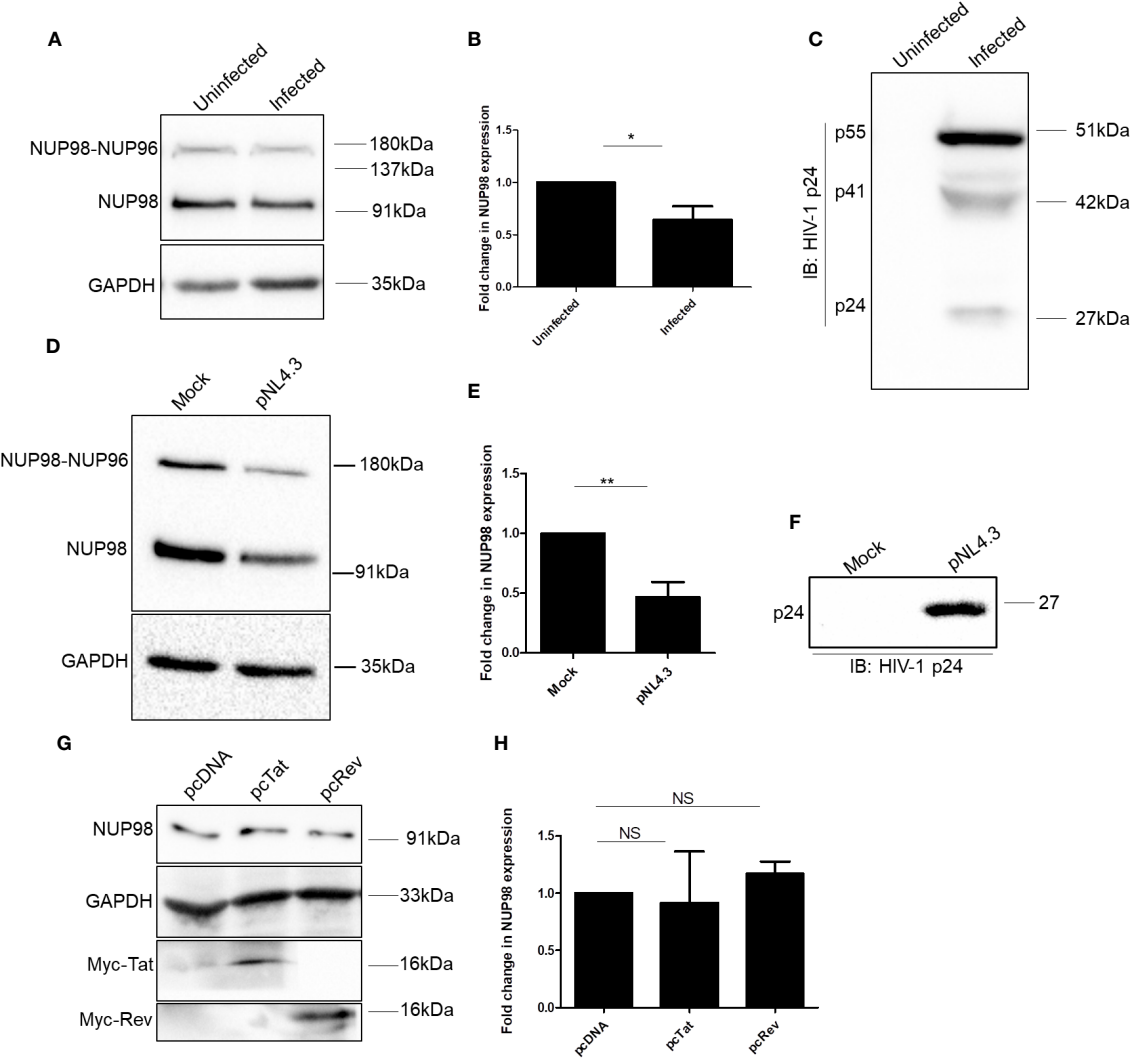
HIV-1 downregulates NUP98 in HEK293T cells. **(A)** Representative Western blot showing endogenous NUP98 levels upon HIV-1 NL4.3 infection of HEK293T cells. Blots were probed with anti-NUP98 and anti-GAPDH antibodies. **(B)** Bar graphs representing the mean fold change of NUP98 expression relative to the uninfected cells from **(A)**. **(C)** Representative Western blot showing intracellular p55 levels upon HIV-1 NL4.3 infection of HEK293T cells. Blots were probed with anti-HIV-1 p24 antibodies. **(D)** Representative Western blot showing endogenous NUP98 levels upon pNL4.3 transfection of HEK293T cells. Blots were probed with anti-NUP98 and anti-GAPDH antibodies. **(E)** Bar graphs representing the mean fold change of NUP98 expression relative to the untransfected cells from **(D)**. **(F)** Representative Western blot showing intracellular p24 levels upon pNL4.3 transfection of HEK293T cells. Blots were probed with anti-HIV-1 p24 antibodies. **(G)** Representative Western blot showing endogenous NUP98 levels in HEK293T cells upon transfection with either pcDNA or pcTat or pcRev. Blots were probed with anti-NUP98, anti-GAPDH, and anti-Myc antibodies. **(H)** Bar graphs representing the mean fold change of endogenous NUP98 expression relative to the vector control from **(G)**. The experiments were performed at least three times. *, *P* < 0.05; **, *P* < 0.01; NS, *P* > 0.05..

To understand the impact of HIV-1 on the NPC components, we checked the expression of some of the NUPs such as NUP98, NUP62, NUP155, NUP133, NUP107, and NUP85 both in SupT1 (CD4^+^) and HEK293T cell lines, 4 days after infection with VSV-G (vesicular stomatitis virus envelope glycoprotein G)-pseudotyped HIV-1 virus (hereafter referred to as HIV-1 NL4.3). We observed that NUP98 protein levels were decreased both in SupT1 and HEK293T cell types upon HIV-1 NL4.3 infection by 4.7- and 1.5-fold, respectively (**Figures 1A, B**, **2A, B**, respectively), whereas the protein levels of other NUPs such as NUP62, NUP155, NUP133, NUP107, and NUP85 remained unaffected (**Supplementary Figures S1A, B**). Infections in these cell types were verified by Western blotting using anti-p24 antibody (**Figures 1C**, **2C**). Speculating that the differences in the mean fold change of NUP98 protein levels between these two cell types may be due to differences in infectivity, the intracellular p55 levels normalized to the corresponding endogenous GAPDH levels were quantified (**Supplementary Figure S1C**). We observed that the percentage of infection by HIV-1 was nearly 50% lower in HEK293T cells as compared with SupT1 cells (**Supplementary Figure S1C**), which indicated that the moderate lowering in NUP98 protein levels upon HIV-1 infection in HEK293T cells was probably due to low infection in this cell type. To further understand how the levels of the NUP98 protein were downregulated by HIV-1 infection, we checked the total mRNA that encodes NUP98 in infected SupT1 and HEK293T cells. In comparison to uninfected cells, we did not observe any significant change in the total NUP98 mRNA upon infection in both cell types (**Supplementary Figure S2A**), suggesting that HIV-1 infection is not affecting either the transcription or stability of NUP98 encoding mRNA in these conditions. This result points to the post-transcriptional regulation of NUP98 levels upon HIV-1 infection through an unknown mechanism(s).

Having observed that NUP98 levels were lowered upon HIV-1 NL4.3 infection, we repeated the experiment to check the protein levels of all the NUPs under study (NUP98, NUP62, NUP155, NUP133, NUP107, and NUP85) in HEK293T cells upon proviral DNA pNL4.3 transfection, which allows the synthesis of the viral genomic RNA and proteins and the release of virions, mimicking the late stages of HIV-1 infection. We found that NUP98 protein levels were significantly downregulated by 2.1-fold in comparison to control cells (**Figures 2D, E**). The expression of viral proteins from the pNL4.3 plasmid upon transfection was verified by Western blotting using an anti-p24 antibody (**Figure 2F**). Furthermore, unlike during infection, we observed downregulation of the protein levels of NUP62 and NUP85 by 1.2- and 2.1-fold, respectively, whereas NUP133 was upregulated by 1.5-fold (**Supplementary Figure S2B**). The NUP85 gene expression results in several transcript variants and isoforms, including 75 kDa and 69 kDa. In our Western blot analysis, we detected two protein bands with the anti-NUP85 antibody corresponding to the 69-kDa and 75-kDa isoforms (**Supplementary Figure S2B**). We quantified the canonical 75-kDa isoform and represented it in the corresponding bar graph (**Supplementary Figure S2B**). The protein levels of other nucleoporins such as NUP155 and NUP107 remained unchanged under transfection conditions (**Supplementary Figure S2B**). Since NUP98 was the only NUP that was downregulated at the protein levels during both transfection in HEK293T and infections in SupT1 and HEK293T cells, we continued our investigations on NUP98. To examine if the viral regulatory factors such as Tat and Rev alone could cause a decrease in the endogenous NUP98 protein levels, we overexpressed these viral factors in HEK293T cells. However, the overexpression of either Tat or Rev in HEK293T cells did not affect the NUP98 protein levels (**Figures 2G, H**), indicating that the downmodulation of NUP98 protein levels by HIV-1 might be Tat- and Rev-independent.

### Overexpression of NUP98 reduces HIV-1 viral protein and transcript levels, decreasing released virus and its infectivity

#### Overexpression of NUP98 reduces intracellular p55 viral protein and viral antigen release (p24 equivalents)

**Figure 3:**
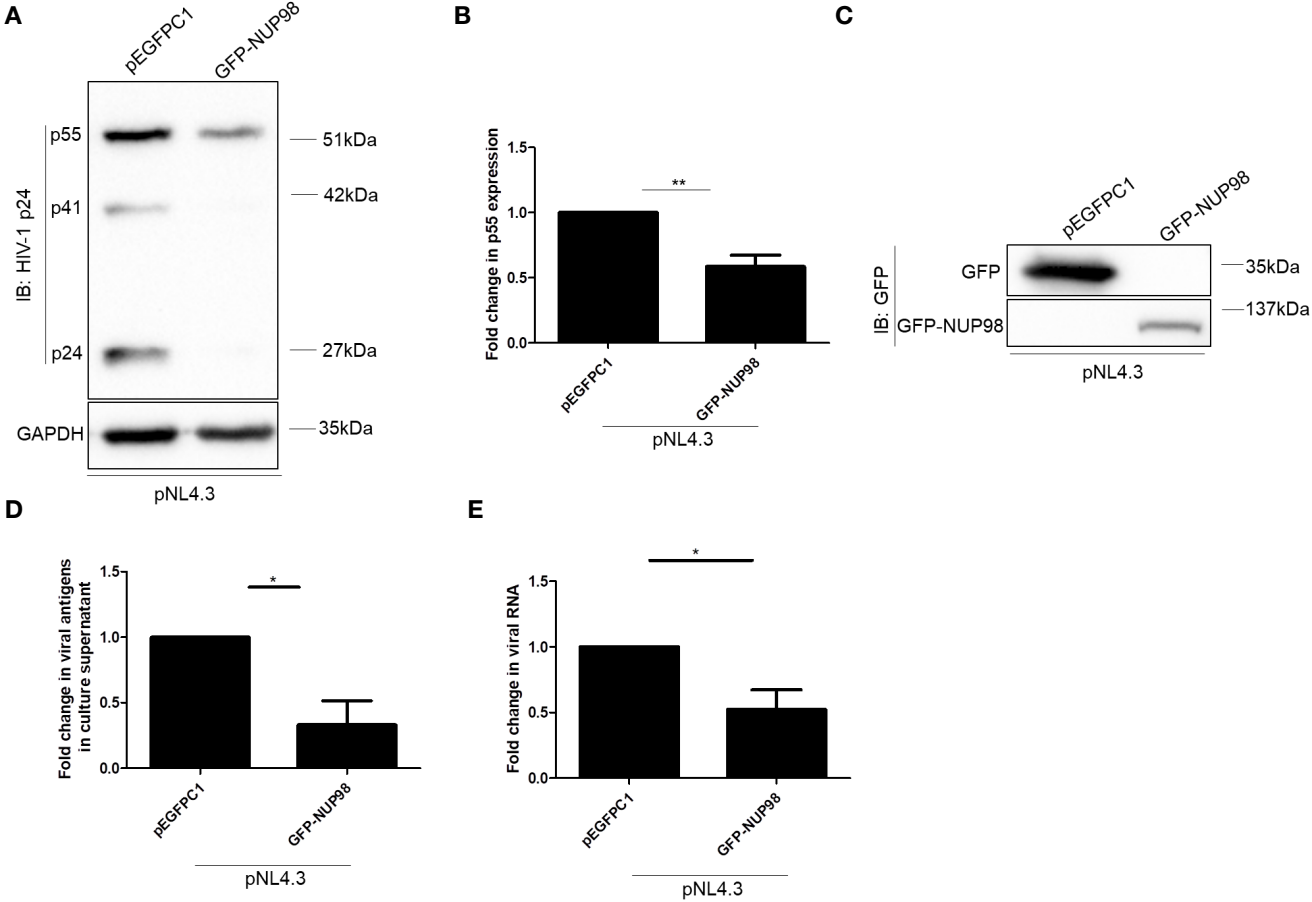
Overexpression of NUP98 reduces HIV-1 p55 protein and RNA levels. **(A–E)** HEK293T cells were co-transfected with pNL4.3 and either pEGFPC1 or GFP-NUP98. Forty-eight hours post-transfection, cells were harvested and the culture supernatant was collected. **(A)** Blots were probed with anti-HIV-1 p24 and GAPDH antibodies. p55 expression was normalized to the loading control GAPDH. **(B)** Bar graphs represent the mean fold change of p55 expression relative to the vector control. **(C)** The GFP-NUP98 expression was verified by Western blotting using the anti-GFP antibody. **(D)** Bar graphs represent the mean fold change of viral antigens in the culture supernatant relative to the vector control. **(E)** Bar graphs represent the mean fold change of intracellular viral RNA relative to the vector control. The experiments were performed at least three times. *, *P* < 0.05; **, *P* < 0.01.

We further investigated the significance of infection-mediated downregulation of NUP98. Toward this, HEK293T cells were co-transfected with GFP-NUP98 and pNL4.3, and we studied the impact of transiently expressed NUP98 on the intracellular levels of the HIV-1 protein p55 (Gag) as well as viral antigen release (p24 equivalents) in the culture supernatant. Western blotting analysis showed that the intracellular levels of p55 were significantly decreased upon overexpression of GFP-NUP98 in comparison to control cells transfected with pEGFPC1 by 1.7-fold (**Figures 3A, B**). The expression of GFP-NUP98 was verified in the cells transfected with the GFP-NUP98 plasmid construct by Western blotting using the anti-GFP antibody (**Figure 3C**). We next estimated the quantity of viral antigens released in the culture supernatant of HEK293T cells co-transfected with GFP-NUP98 and pNL4.3 by p24 ELISA. In agreement with the cellular viral protein p55 levels, viral antigen in the culture supernatants of HEK293T cells transfected with GFP-NUP98 was attenuated by 3-fold compared with the control cells (**Figure 3D**). The trypan blue dye exclusion assay showed that transient overexpression of NUP98 had a negligible effect on cell viability (**Supplementary Figure S3**). We then checked if any of the other NUPs under this study could also affect the HIV-1 virion production in general. Therefore, as another FG-rich NUP, the NUP62’s role on intracellular p55 protein expression and viral antigen release in the culture supernatant was evaluated. Upon co-transfection with pNL4.3 and GFP-NUP62, we observed no significant effect on either intracellular p55 protein expression or viral antigen release by transiently expressed GFP-NUP62 (**Supplementary Figures S4A–C**). The expression of GFP-NUP62 was verified in the cells transfected with the GFP-NUP62 plasmid construct by Western blotting using the anti-GFP antibody (**Supplementary Figure S4D**). Thus, these data suggest that inhibition of HIV-1 protein expression was NUP98-specific in our conditions with respect to the selected NUPs that we were studying.

#### Overexpression of NUP98 reduces viral transcript levels

**Figure 4:**
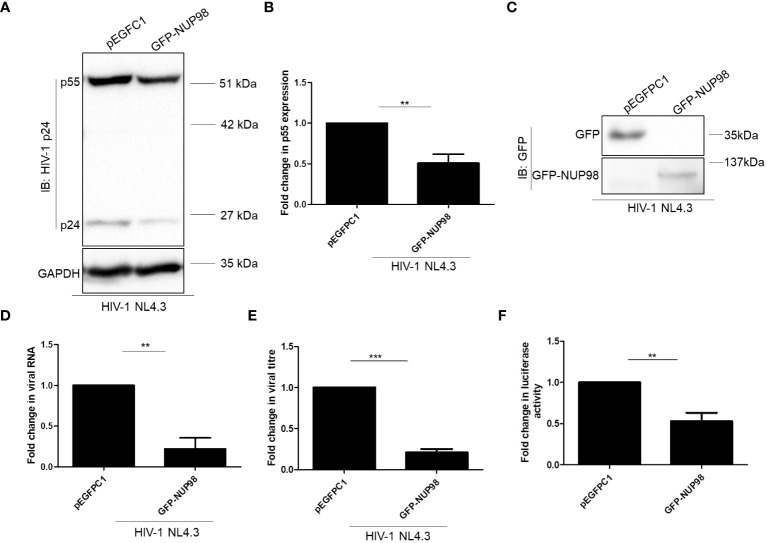
Overexpression of NUP98 reduces viral protein and RNA levels during HIV-1 NL4.3 infection in HEK293T cells. **(A–E)** HEK293T cells were transfected with either pEGFPC1 or GFP-NUP98. Twenty-four hours post-transfection, cells were infected with HIV-1 NL4.3, and 48 hpi cells were harvested either for lysate preparation or RNA isolation, and the culture supernatant was collected for p24 ELISA. **(A)** Blots were probed with anti-HIV-1 p24 and anti-GAPDH antibodies. p55 expression was normalized to the loading control GAPDH. **(B)** Bar graphs represent the mean fold change of p55 expression relative to the vector control. **(C)** The GFP-NUP98 expression was verified by Western blotting using the anti-GFP antibody. **(D)** Bar graphs represent the mean fold change of viral RNA expression relative to the vector control. **(E)** Bar graphs represent the mean fold change of viral antigens relative to the vector control. **(F)** HEK293T cells were co-transfected with pNL4.3 and either pEGFPC1 or GFP-NUP98. Forty-eight hours post-transfection, culture supernatants were collected. The culture supernatant containing the virus (p24 equivalents) was used to infect TZM-bl cells. Forty-eight hours post-infection, cells were harvested for luciferase assay. Bar graphs represent the mean fold change of luciferase activity relative to the vector control. The experiments were performed at least three times. **, *P* < 0.01; ***, *P* < 0.001.

The decrease in both intracellular p55 levels and viral antigen release led us to speculate if NUP98 actually reduced total viral RNA. To this end, HEK293T cells were co-transfected with GFP-NUP98 and pNL4.3 as mentioned above. After 48 h of transfection, cells were harvested and total viral RNA was quantified by qPCR using the primers listed in **Table 1**. Indeed, we observed that the overexpression of NUP98 reduced total viral transcript (Env mRNA) levels (**Figure 3E**). To test if NUP98-mediated reduction of viral p55, RNA levels, and viral antigen release was also true for cells infected with HIV-1 NL4.3, HEK293T cells were first transfected either with pEGFPC1 or GFP-NUP98 for 24 h and then infected with HIV-1 NL4.3 for 48 h. As expected, even under these infection conditions, intracellular p55 levels were reduced by GFP-NUP98 (**Figures 4A, B**). The expression of GFP-NUP98 was verified in the cells transfected with the GFP-NUP98 plasmid construct by Western blotting using the anti-GFP antibody (**Figure 4C**). In addition, viral RNA levels and viral antigen release were also reduced upon NUP98 transient overexpression (**Figures 4D, E**). The reduced total viral RNA upon NUP98 overexpression, thus, explains the decrease in the intracellular p55 levels and viral antigen release. It should be further noted that the quantification of total viral RNA by RT-qPCR would only infer the levels of RNA at a given state, and therefore, we cannot rule out the possible combined effect of viral transcription inhibition and decrease in viral transcript stability by NUP98 during HIV-1 infection. We also extended our study to see if transiently expressed GFP-NUP98 could affect p55 levels from another proviral construct pIndie-C1 (subtype C) (**Supplementary Figures S5A, B**). pIndie-C1 is an infectious molecular clone isolated from the HIV-1 subtype C strain 93IN101 of pandemic potential, which is prevalent in India (51). In line with the data obtained from pNL4.3 (subtype B) (**Figures 3A, B**), p55 levels from pIndie-C1 were significantly reduced by 5-fold upon transient expression of NUP98 (**Supplementary Figures S5A, B**). Hence, it can be inferred that NUP98 negatively affected viral protein levels both in subtypes B and C of HIV-1. The expression of GFP-NUP98 was verified in the cells transfected with the GFP-NUP98 plasmid construct by Western blotting using the anti-GFP antibody (**Supplementary Figure S5C**). Taken together, these results suggest that NUP98 negatively impacts viral RNA levels.

#### Virions released from NUP98 overexpressing producer cells showed reduced infectivity

As we observed that NUP98 attenuated the intracellular p55, viral RNA, and viral antigens released in the culture supernatant from the producer cell, we reasoned whether NUP98 overexpression might also affect the infectivity of the HIV-1 particles released from the producer cell. Toward this, HIV-1 was prepared from HEK293T cells by co-transfecting with the plasmids pNL4.3 and GFP-NUP98. As a control, cells were also co-transfected with pNL4.3 and pEGFPC1. The equal amounts of viral particles (5 ng/ml of p24 equivalents) produced from either control cells or NUP98 overexpressing cells were then used to infect the target reporter cell line TZM-bl. The TZM-bl cell line is genetically engineered from the HeLa cell line and stably expresses the receptor (CD4) and co-receptor (CCR5). The genome of this cell line also harbors separate integrated copies of reporter genes such as luciferase and β-galactosidase, and their expression is under the control of the HIV-1 promoter LTR (58). After 48 h post-infection, luciferase activity driven by the HIV-1 LTR promoter was evaluated. To our surprise, the infectivity of the virus prepared from the producer cells (HEK293T) expressing NUP98 was decreased by 2-fold in comparison to the control cells (**Figure 4F**).

### Knockdown of NUP98 enhances HIV-1 viral protein and transcript levels, increasing the released virus and its infectivity

**Figure 5:**
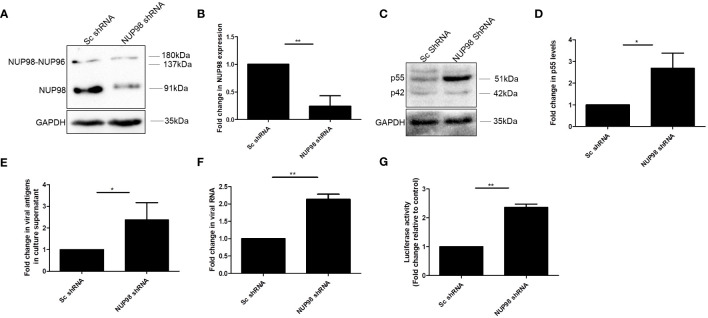
Knockdown of NUP98 in SupT1 cells ameliorates HIV-1 viral protein, RNA levels, and infectivity. **(A–G)** SupT1 cells were infected with HIV-1 NL4.3 for 24 h and then transduced with lentivirus containing either control scrambled (Sc) shRNA or NUP98 shRNA. Seventy-two hours post-transduction, cells were harvested either for lysate preparation or RNA isolation, and culture supernatants were collected for p24 ELISA and for the TZM-bl reporter assay. **(A)** Blots were probed with anti-NUP98 and anti-GAPDH antibodies. NUP98 expression was normalized to the corresponding loading control GAPDH. **(B)** Bar graphs represent the mean fold change of NUP98 expression relative to Sc shRNA. **(C)** Using the same lysates as in **(A)**, blots were probed with anti-p24 and anti-GAPDH antibodies. p55 expression was normalized to the loading control GAPDH. **(D)** Bar graphs represent the mean fold change of p55 expression relative to Sc shRNA. **(E)** Bar graphs represent the mean fold change of viral antigens relative to Sc shRNA **(F)** Bar graphs represent the mean fold change of viral RNA expression relative to Sc shRNA. **(G)** Equal amounts of the virus (p24 equivalents) collected from shRNA-transduced cells (Sc shRNA and NUP98 shRNA) were used to infect TZM-bl cells. Forty-eight hours post-infection, cells were harvested for luciferase assay. Bar graphs represent the mean fold change of luciferase activity relative to the Sc shRNA control. The experiments were performed at least three times. *, *P* < 0.05; **, *P* < 0.01.

To corroborate the effect of NUP98 on HIV-1 replication, the endogenous NUP98 in SupT1 cells was depleted using shRNA. Previously, it was shown that NUP98 promotes the integration of HIV-1 and that the depletion of NUP98 led to decreased infectivity (22). To surpass the effect of NUP98 depletion on the integration of HIV-1 DNA, SupT1 cells were first infected with HIV-1 NL4.3 for 24 h and then transduced with lentivirus containing shRNA that targeted NUP98. Seventy-two hours post-transduction, cells were harvested to analyze the intracellular p55 protein and viral RNA levels by Western blotting and RT-qPCR, respectively, and culture supernatants were collected to measure the virus-associated p24 by ELISA. Western blotting analysis showed that the endogenous NUP98 was significantly downregulated in comparison to cells transduced with scrambled (Sc) shRNA (**Figures 5A, B**). The viability of the cells that were depleted of NUP98 was assessed by trypan blue dye exclusion assay, and we observed that depletion of NUP98 had a negligible effect on the cell viability in comparison to control cells (**Supplementary Figure S6**). In agreement with overexpression studies in HEK293T cells, the depletion of endogenous NUP98 enhanced the intracellular p55 levels as well as the virus-associated p24 in the culture supernatant (**Figures 5C–E**). Moreover, viral RNA levels were also increased upon depletion of NUP98, suggesting that NUP98 plays an important antiviral role in the HIV-1 gene expression (**Figure 5F**). To examine the infectivity of the virus that emerged from SupT1 cells depleted of NUP98, TZM-bl cells were infected with equal amounts of viral particles (5 ng/ml of p24 equivalents) produced from either NUP98 shRNA- or Sc shRNA-transduced SupT1 cells as previously described. We observed that the infectivity of the virus produced from NUP98-depleted cells was enhanced (**Figure 5G**).

Thus, with the overexpression and knockdown studies, we conclude that NUP98 reduced intracellular viral RNA, viral p55 levels, released virus titers, and the infectivity of the released virus.

### NUP98 is associated with HIV-1 LTR and decreases HIV-1 LTR-driven gene expression

**Figure 6:**
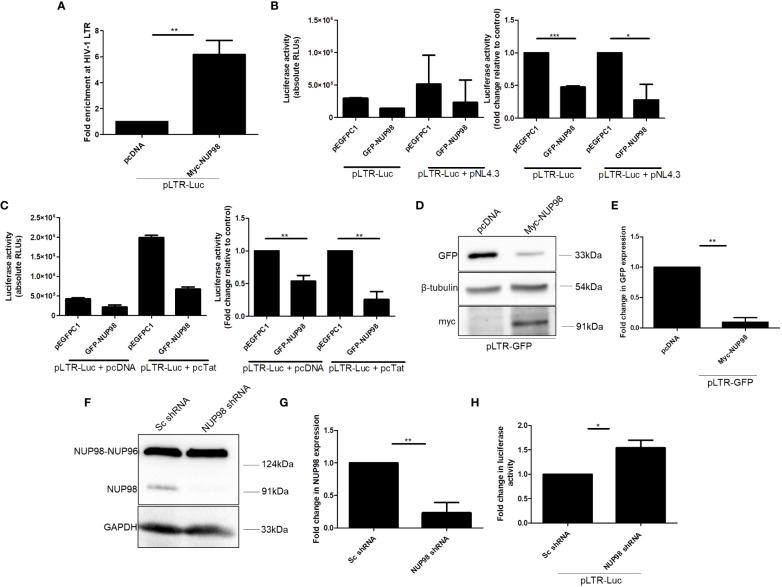
NUP98 associates with HIV-1 LTR and diminishes HIV-1 LTR-driven transcription. **(A)** HEK293T cells were co-transfected with pLTR-Luc and either pcDNA or Myc-NUP98. Forty-eight hours post-transfection, cells were subjected to cross-linking, and the ChIP assay was performed with the anti-Myc antibody. The pull-down fractions were analyzed by qPCR using primers against HIV-1 LTR. Bar graphs represent the mean fold enrichment of Myc-NUP98 at HIV-1 LTR relative to the vector control. **(B)** HEK293T cells were co-transfected with pLTR-Luc and either pEGFPC1 or GFP-NUP98 in the presence or absence of pNL4.3. Forty-eight hours post-transfection, cells were lysed in reporter lysis buffer and lysate was used for luciferase activity. Bar graphs represent the mean basal luciferase activities (**B**, left panel). Bar graphs represent the mean fold change of luciferase activities in relative vector control (**B**, right panel). **(C)** HEK293T cells were co-transfected with pLTR-Luc, pcDNA, or pcTat and either pEGFPC1 or GFP-NUP98. Forty-eight hours post-transfection, cells were lysed in reporter lysis buffer and lysate was used for luciferase activity. Bar graphs represent the mean basal luciferase activities (**C**, left panel). Bar graphs represent the mean fold change of luciferase activity relative to the vector control (**C**, right panel). **(D, E)** HEK293T cells were co-transfected with pLTR-GFP and either pcDNA or Myc-NUP98. Forty-eight hours post-transfection, cells were lysed and lysate was used for Western blotting. **(D)** Blots were probed with antibodies against GFP, β-tubulin, and myc. GFP expression was normalized to the loading control β-tubulin. **(E)** Bar graphs represent the mean fold change of GFP expression relative to the vector control. **(F, G)** HEK293T cells were transfected with NUP98 targeting shRNA. As a control, cells were also transfected with scrambled (Sc) non-specific shRNA. **(F)** Blots were probed with anti-NUP98 and anti-GAPDH antibodies. The expression of NUP98 was normalized to the loading control GAPDH. **(G)** Bar graphs represent the mean fold change of NUP98 expression relative to the Sc shRNA control. **(H)** Forty-eight hours post-transfection with shRNAs, cells were again transfected with pLTR-Luc and incubated further for 24 h. Cells were lysed in reporter lysis buffer and lysate was used for luciferase activity. Bar graphs represent the mean fold change of luciferase activity relative to the Sc shRNA control. The experiments were performed at least three times. *, *P* < 0.05; **, *P* < 0.01.

As overexpression and knockdown studies indicated that NUP98 reduced both viral protein and RNA levels, we investigated if NUP98 affected the HIV-1 LTR-driven transcription by associating with HIV-1 LTR. To check this hypothesis, HEK293T cells were co-transfected with Myc-NUP98 and plasmid construct containing the full-length HIV-1 LTR promoter (pLTR-Luc), and 48 h later, ChIP-qPCR was performed using the anti-Myc antibody. For the qPCR analysis, the primers were designed to amplify the region toward the end of 5'LTR of HIV-1, i.e., +68 nt to +168 nt, where +1 nt indicates the transcription start site in the LTR promoter (**Table 1**) (59). We found that transiently expressed NUP98 was enriched at HIV-1 LTR by 6-fold in comparison to the vector control (**Figure 6A**). Having confirmed that NUP98 is associated with HIV-1 LTR, we next investigated its impact on HIV-1 LTR-driven transcription. Toward this, we used the pLTR-Luc construct that expresses the luciferase gene from the HIV-1 LTR as a reporter system. HEK293T cells were co-transfected with pLTR-Luc and pEGFPC1 or GFP-NUP98 in the absence or presence of pNL4.3. All HIV-1 viral proteins were provided in the form of the molecular clone pNL4.3 to mimic the condition wherein the possible regulation of LTR activity by viral proteins including HIV-1 Tat, which is the main viral transcription regulator of LTR promoter, would be ensured. We indeed observed that the transiently expressed GFP-NUP98 decreased the expression of the luciferase gene from HIV-1 LTR in comparison to the vector control, irrespective of whether pNL4.3 was provided or not (**Figure 6B**). These results indicate that NUP98 prevents basal viral gene expression from the HIV-1 LTR promoter. To further understand if the inhibition of basal transcription of HIV-1 LTR by NUP98 could be rescued by HIV-1 Tat, HEK293T cells were co-transfected with pLTR-Luc and pcDNA Tat or pcDNA along with GFP-NUP98 or pEGFPC1. We observed that NUP98 reduced the luciferase activity by 3.9-fold even in the presence of Tat, indicating that Tat could not rescue the NUP98-mediated downregulation of HIV-1 LTR activity (**Figure 6C**). We further substantiated these observations using the pLTR-GFP construct which retains LTRs, genes for early viral regulatory proteins, Tat, and Rev, but lacks genes for Env, Gag, Gag-Pol, Nef, Vif, and Vpr. In place of gag, pol, vif, and vpr, GFP was inserted such that its expression is directly under the control of LTR. The expression of GFP from the LTR promoter was then analyzed by Western blotting using the anti-GFP antibody and normalized to the loading control β-tubulin. Even under these conditions, NUP98 was able to reduce the GFP expression driven by HIV-1 LTR by 10-fold (**Figures 6D, E**). To further validate if the negative effect of NUP98 on HIV-1 LTR was indeed HIV-1 LTR-specific, we co-transfected both Myc-NUP98 or pcDNA and pEGFPC1 [GFP expression from the cytomegalovirus (CMV) promoter] into HEK293T cells. The expression of GFP from the CMV promoter was then analyzed by Western blotting using the anti-GFP antibody and normalized to the loading control β-tubulin. However, the overexpression of NUP98 did not change the levels of GFP expressed from the CMV promoter (**Supplementary Figures S7A, B**). It should be noted that since the CMV promoter in the pEGFPC1 construct does not contain all the elements of the CMV, we cannot exclude the possible inhibitory effect of NUP98 on CMV-driven gene expression in the context of CMV infection. Similarly, we also tested if NUP98 could affect the SIV promoter by co-transfecting HEK293T cells with pSIV_AGM_-Luc-R^−^E^−^Δvif and GFP-NUP98 and measuring SIV promoter-driven luciferase activity. We found that NUP98 had no influence on the SIV LTR promoter (**Supplementary Figure S7C**), suggesting that NUP98 specifically suppresses HIV-1 LTR activity.

To corroborate the role of NUP98 in the regulation of HIV-1 LTR-driven transcription, the expression of NUP98 was depleted in HEK293T cells by transfecting the cells with the target shRNA plasmid. As a control, cells were also transfected with scrambled (Sc) shRNA plasmid independently. The significant depletion of the NUP98 protein was observed in the cells transfected with shRNA targeting NUP98 but not in the cells transfected with Sc shRNA (**Figures 6F, G**). Forty-eight hours after transfection with shRNAs, cells were retransfected with pLTR Luc. After 24 h of incubation, cells were harvested and luciferase activity was performed. The data from luciferase assays showed that the HIV-1 LTR activity was increased by 1.5-fold in the cells depleted of NUP98 in comparison to the cells transfected with Sc shRNA, corroborating that NUP98 decreased the transcription from the HIV-1 LTR promoter (**Figure 6H**). These data, thus, provide evidence that NUP98 specifically suppressed the HIV-1 LTR-driven viral basal gene expression.

### The NUP98-mediated decrease in viral gene expression is dependent on the NRE region of HIV-1 LTR

**Figure 7:**
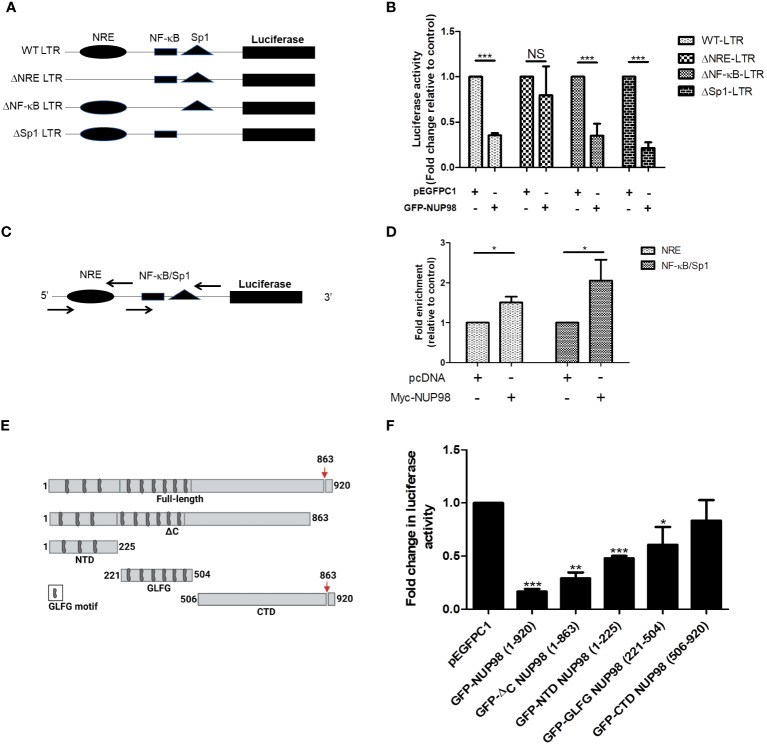
The NRE of LTR is required for NUP98-mediated perturbation of HIV-1 LTR-directed gene expression. **(A)** The schematic representation of the full-length wild-type (WT) LTR and its deletion mutant plasmid constructs. **(B)** HEK293T cells were co-transfected either with pEGFPC1 or GFP-NUP98 and pLTR-Luc or LTR deletion mutant plasmid constructs. Forty-eight hours post-transfection, cells were lysed in reporter lysis buffer and lysate was used for luciferase activity. Bar graphs represent the mean fold change of luciferase activities of LTR constructs in the presence of the GFP-NUP98 relative vector control. **(C)** The schematic representation of primers that amplify NRE and NF-κB/Sp1 binding sites. **(D)** HEK293T cells were co-transfected with either pcDNA or Myc-NUP98 and pLTR-Luc. Forty-eight hours post-transfection, cells were harvested for the ChIP-qPCR assay. The immunoprecipitated DNA was used as a template for qPCR using the primers that amplify NRE and NF-κB/Sp1 binding sites. Bar graphs represent the mean fold enrichment of NUP98 at NRE and NF-κB/Sp1 binding sites relative to the vector control. **(E)** Schematic representation of the full-length and the domains of NUP98; the down arrow indicates the autoproteolytic cleavage site. **(F)** HEK293T cells were co-transfected with pLTR-Luc, pcTat, and plasmids expressing the full-length and the domains of NUP98. Forty-eight hours post-transfection, cells were lysed in reporter lysis buffer and lysate was used for luciferase activity. Bar graphs represent the mean fold change of luciferase activity relative to the vector control. The experiments were performed at least three times. One-way ANOVA with Tukey’s multiple comparisons test was used for statistical analysis for figure B using GraphPad Prism. *, *P* < 0.05; **, *P* < 0.01; ***, *P* < 0.001; NS, *P* > 0.05.

The HIV-1 LTR promoter is functionally divided into the negative regulatory element (NRE), enhancer, core, and TAR regions (4). The NRE of LTR was known to downmodulate the LTR-directed HIV-1 gene expression (4). While the enhancer region contains binding sites for transcription factors such as nuclear factor-kappa B (NF-κB), the core region harbors the binding sites for constitutive transcription factors such as specificity protein 1 (Sp1) and TATA-box binding protein (TBP). As the above experiments suggested that NUP98 negatively affected the basal viral gene expression, which was independent of Tat, we hypothesized that the elements upstream to the TAR region might play a regulatory role in the NUP98-mediated lowering of viral gene expression. To test this hypothesis, we created deletion mutants of HIV-1 LTR in the pLTR-Luc construct, lacking NRE or binding sites for NF-κB and Sp1 (**Figure 7A**). HEK293T cells were co-transfected with these deletion mutants and GFP-NUP98 or pEGFPC1. The comparison of basal luciferase activities of these LTR deletion mutants with that of WT LTR in cells transfected with pEGFPC1 suggested that the deletion mutants that lacked NF-κB and Sp1 binding sites significantly lost the promoter activity, suggesting that the binding sites for these transcription factors are important for the basal activity of the LTR promoter (**Supplementary Figure S8**). In contrast, deletion of the NRE region (ΔNRE LTR) did not affect basal transcription activity (**Supplementary Figure S8**). We further normalized the luciferase activities for each LTR construct, including WT LTR, in the presence of GFP-NUP98 with the luciferase activities measured in the background of the corresponding vector control pEGFPC1. Consistent with the above experiments, the negative effect of NUP98 on WT LTR activity was evident relative to the vector control (pEGFPC1) (**Figure 7B**). Moreover, the promoter activity of LTR deletion mutants such as ΔNF-κB and ΔSp1 was also shown to be suppressed by NUP98 relative to the vector control, suggesting that these elements of LTR may not be required for NUP98-mediated suppression of HIV-1 gene expression (**Figure 7B**). Interestingly, the NUP98-mediated suppressing effect on the luciferase activity from the ΔNRE LTR construct was not observed (**Figure 7B**), pointing to this region being involved in NUP98-mediated LTR activity suppression. We next performed ChIP-qPCR using specific primers (**Table 1**) to amplify specific regions of LTR (**Figure 7C**) to study the occupancy of Myc-NUP98. We observed the enrichment of Myc-NUP98 on both the NRE and NF-κB/Sp1 regions (**Figure 7D**). However, considering that ΔNF-κB or ΔSp1 did not affect NUP98-mediated suppression of LTR activity, we conclude that NUP98-mediated lowering of viral gene expression is dependent on the NRE region of HIV-1 LTR. It should be noted that this does not rule out the possibility of the involvement of NF-κB and Sp1 in NUP98-mediated regulation of HIV-1 LTR activity. This observation further warrants future investigation to understand the molecular events underlying the NRE-specific suppressive effect of NUP98 on HIV-1 gene expression.

### The N-terminal region of NUP98 (1–504) contributes to NUP98-mediated inhibition of HIV-1 LTR-driven transcription

Next, we deliberated on understanding the contribution of different domains of NUP98 in the regulation of HIV-1 LTR-driven transcription. The constructs that express domains of NUP98 were previously described and schematically shown in **Figure 7E** (14). HEK293T cells were co-transfected with pLTR Luc, pcDNA Tat, and pEGFPC1 or NUP98 GFP or plasmid expressing different domains of NUP98. As observed in previous experiments, the full-length NUP98 (1–920) reduced the promoter activity of HIV-1 LTR (**Figure 7F**). A similar effect was also observed for GFP-ΔC NUP98 (1–863), which has a 6-kDa region removed from the C-terminal end of NUP98 (**Figure 7F**). The GFP-NTD NUP98 (1–225), which expressed only the N-terminal domain of NUP98, was also found to reduce the HIV-1 LTR-driven activity by 2-fold (**Figure 7F**). When the reduction in the HIV-1 LTR-driven activities was calculated for GFP-GLFG (glycine-leucine-phenylalanine-glycine) NUP98 (221–504) and GFP-CTD NUP98 (506–920), representing the GLFG domain and the C-terminal domain, respectively, we found that the GLFG domain reduced the LTR activity significantly by 1.6-fold, whereas the CTD of NUP98 did not affect the LTR activity (**Figure 7F**). The suppression of HIV-1 LTR gene expression by both NTD and GLFG domains indicates that the N-terminal region spanning both these domains (1–504) is required for NUP98 to downregulate HIV-1 LTR activity, with the NTD playing the dominant role.

Overall, we infer that NUP98, which was downregulated upon HIV-1 infection, functions as a negative regulator of HIV-1 LTR-driven transcription, consequently lowering released virions. Interestingly, the virus generated from the producer cells transiently expressing NUP98 showed lower infectivity, while the virus generated from NUP98 knockdown CD4^+^ T cells showed higher infectivity, warranting further investigations on the antiviral properties of NUP98.

## Discussion

In this study, we examined the expression levels of NUPs, each representing a subcomplex in NPC, during the late stages of HIV-1 infection and demonstrated a non-canonical antiviral role of NUP98 in HIV-1 infection. We observed a decrease in the levels of the NUP98 protein during HIV-1 infection in both SupT1 and HEK293T cell lines (**Figures 1A**, **2A**). In addition, the precursor form, i.e., NUP98–NUP96, also seemed to be downregulated, which was readily observed in HEK293T cells transfected with pNL4.3 (**Figure 2D**) and less clearly in HEK293T cells infected with HIV-1 NL4.3 (**Figure 2A**). Since the precursor NUP98–NUP96 levels at the basal state in SupT1 cells were below detectable (**Figure 1A**), we cannot conclude the effect of HIV-1 infection on this precursor form in SupT1 cells. As we have described earlier, the products of NUP98 gene expression could be either the NUP98 protein alone (expressed from alternatively spliced short mRNA) or the NUP98 and NUP96 protein precursor (expressed from longer mRNA and undergoes autoproteolytic cleavage to give rise to NUP98 and NUP96). Given this versatile regulation of NUP98 gene expression, we deduce that HIV-1 may reduce both the NUP98 protein and its precursor levels by affecting any of these processes either alone or in combination.

Several host factors were earlier shown to associate with and regulate HIV-1 LTR (50, 60–67). Indeed, the NUPs such as NUP153 and TPR were shown to associate with HIV-1 LTR and enhance viral gene expression (67). Although this previous report showed the association of NUP98 with HIV-1 LTR, the significance of this association in the context of viral gene expression or infectivity was not explored. In agreement with these studies, we also showed that NUP98 associates with the HIV-1 LTR promoter. Given the observation that NUP98 associates and activates several host gene promoters, it will be intriguing to check how NUP98 could be recruited to the HIV-1 LTR promoter (68). Concordantly, through overexpression and knockdown studies, we showed that NUP98 inhibits the HIV-1 gene transcription from the promoter HIV-1 LTR. To understand the underlying mechanism of LTR-directed gene expression inhibition by NUP98, we constructed LTR promoter mutants and found that NUP98 relied on NRE of HIV-1 LTR to exhibit its suppressive effect on HIV-1 gene expression. Furthermore, we showed that NUP98 occupies the NRE region and requires the N-terminal region (1–504) to suppress the HIV-1 LTR-driven gene expression.

We further checked if transiently expressed NUP98 could interact with some of the well-established host protein regulators of HIV-1 LTR such as NF-κB (p65), HEXIM1, and HDAC1. The Western blotting analysis of immunoprecipitated complexes showed that none of these factors interacted with transiently expressed NUP98 (**Supplementary Figures S9A, B**). Although a previous study showed that fused NUP98 proteins, i.e., NUP98-HOXA9 and NUP98-PMX1, interact with HDAC1 and are involved in the regulation of genes implicated in acute leukemia (46), our experiments with transiently expressed NUP98 indicate that unfused NUP98 might not interact with HDAC1. Nevertheless, based on co-IP analysis with transiently expressed NUP98, we cannot rule out the functional interaction between endogenous NUP98 and NF-κB, HEXIM1, or HDAC1. Future investigations will be required to understand the possible physical interaction between NUP98 and members of protein complexes that regulate HIV-1 gene expression.

It is well established that many of the host co-factors as well as restriction factors are packaged into the released HIV-1 virions and that these factors decide the fate of the virus in the target cells by employing a wide range of mechanisms (36, 54). For instance, the APOBEC family member APOBEC3G, by being packaged into the released viral particles, was shown to cause fatal mutations in the viral genome during the reverse transcription step of the viral life cycle and, thus, limit the infectivity of the virus during new rounds of infection in a target cell (36). Since we observed that NUP98 reduced the infectivity of released viral particles, we checked if NUP98 is packaged into the emerging virions from the producer cells (SupT1 and HEK293T). We probed the presence of both endogenous (from SupT1 and HEK293T) and transiently (from HEK293T) expressed NUP98 in the virion particles released from these producer cells. We found that the endogenous NUP98 was not packaged into the released virion particles produced either from SupT1 or HEK293T cells (**Supplementary Figures S10A, B**). We further examined if the transiently overexpressed NUP98 could be packaged into the virion particles. However, owing to the suppressive effect of NUP98 on intracellular p55 levels, the viral particles released were very low to be detected by Western blotting. Thus, it is difficult to conclude whether transiently expressed NUP98 could be packaged into the released virions under these conditions (**Supplementary Figure S10C**).

To keep pace with the virus, some of the host proteins gain antiviral functions (reviewed in (36)). Most of these factors are frequently induced by interferon (IFN) signaling in response to viral infections (36, 69). Several reports demonstrated that NUP98 is an interferon-inducible protein and is implicated as an antiviral factor for viruses including poliovirus, cardiovirus, and influenza virus (40, 70–74). In these cases, the NUP98 protein was shown to be targeted during viral infection by several mechanisms including phosphorylation, degradation, or specific cleavage, thus reducing intracellular NUP98 levels (71–74). Recently, SARS-CoV-2-encoded Orf6 has been shown to target the NUP98, which results in the inhibition of the import of signal transducer and activator of transcription (STAT) and thereby antagonizes the interferon signaling pathway (70). HIV-1 employs several mechanisms to counteract host restriction factors that prevent different steps of viral replication (36). The best-known restriction factor to be targeted by HIV-1 is the APOBEC family member APOBEC3G. Using its accessory proteins such as Vif and Vpr, HIV-1 targets and reduces intracellular APOBEC3G through multiple mechanisms (36). Given a plethora of these counter mechanisms exhibited by viruses of different families including HIV-1, it is reasonable to assume that HIV-1 by employing yet unknown mechanism(s) reduced the NUP98 protein levels for the benefit of its replication.

Based on the evidence presented in the manuscript, we conclude that NUP98, which is conventionally involved in the transport of molecules as a member of the nuclear pore complex, non-canonically functions as an anti-HIV-1 factor through two different mechanisms: 1) limiting the viral gene transcription through interaction with HIV-1 LTR and 2) lowering the infectivity of the virus released from a producer cell. The downregulation of this antiviral factor during infection can be a host restriction evasion strategy employed by HIV-1. We believe that more insights into the understanding of molecular events underlying NUP98-mediated HIV-1 gene expression repression will help us understand the complex biology behind the host–HIV-1 conflicts.

## Data availability statement

The original contributions presented in the study are included in the article/**Supplementary Material**. Further inquiries can be directed to the corresponding author.

## Author contributions

KC: Formal analysis, Investigation, Methodology, Writing – original draft, Writing – review & editing, Conceptualization, Data curation. SY: Formal analysis, Investigation, Methodology, Writing – original draft, Writing – review & editing. WF: Investigation, Methodology, Writing – original draft, Writing – review & editing, Formal analysis. CK: Formal analysis, Investigation, Methodology, Writing – original draft, Writing – review & editing. SS: Formal analysis, Investigation, Methodology, Writing – original draft, Writing – review & editing. KM: Conceptualization, Formal analysis, Funding acquisition, Investigation, Methodology, Writing – original draft, Writing – review & editing. SB: Conceptualization, Data curation, Formal analysis, Funding acquisition, Investigation, Methodology, Project administration, Supervision, Writing – review & editing, Writing – original draft.
