# Supplementary material for: The nuclear pore protein NUP98 impedes LTR-driven basal gene expression of HIV-1, viral propagation, and infectivity
Source: Front Immunol. 2024 Feb 21;15:1330738. doi: 10.3389/fimmu.2024.1330738 (PMC10914986; doi:10.3389/fimmu.2024.1330738)
Supplement: Supplementary file 1 [file DataSheet_1.docx]

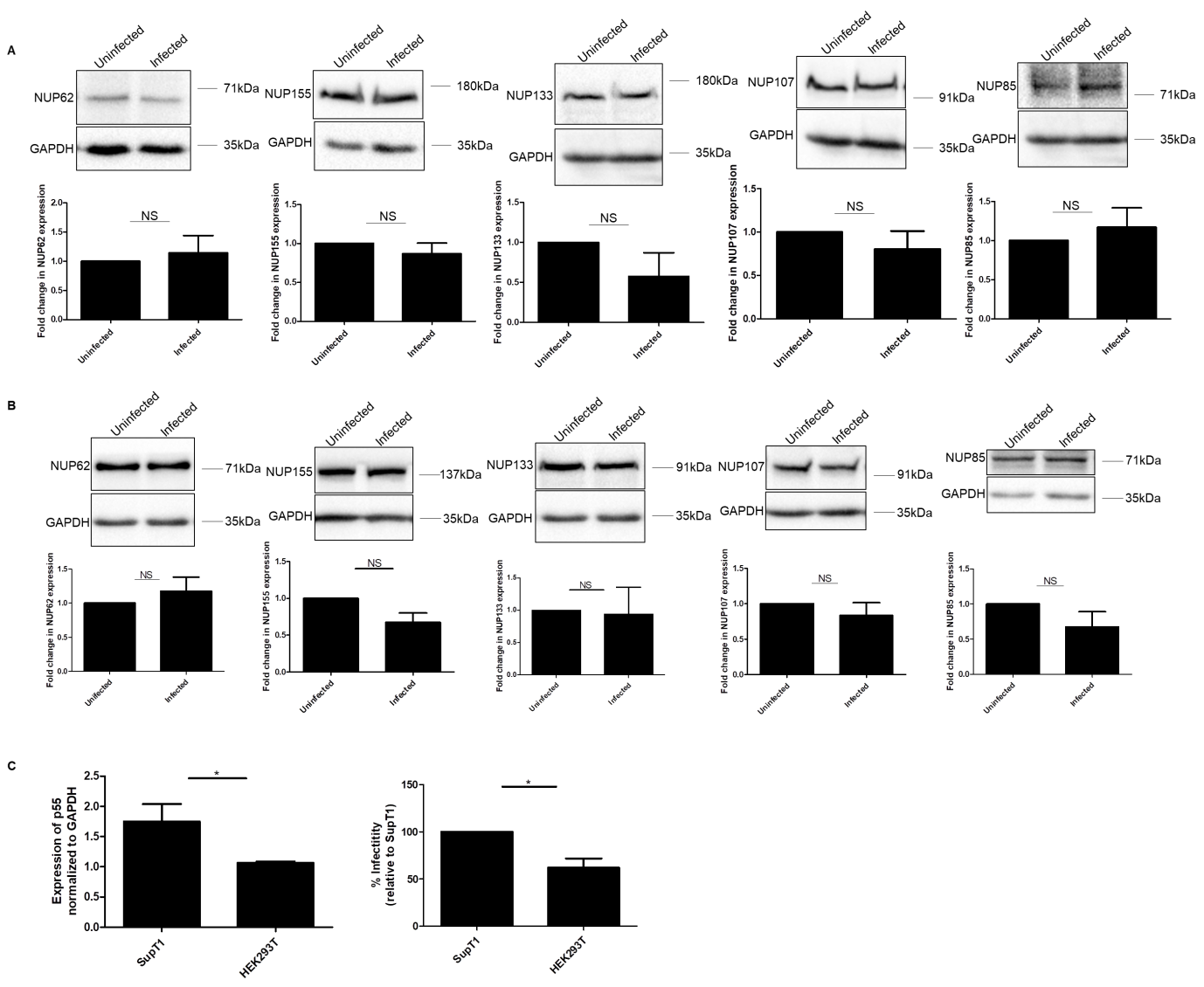


**Figure S1**. Expression of NUPs during HIV-1 infection in SupT1 and HEK29T cells. **(A, B)** Cells (SupT1, A; HEK293T, B) were infected with HIV-1 NL4.3 virus. Four days after infection, the cells were lysed and the lysate was used for western blotting. Blots were probed with anti-NUP62, NUP155, NUP133, NUP107, NUP85 and anti-GAPDH antibodies (upper panels A, B). The expression of NUPs was normalized to the corresponding loading control, GAPDH. Bar graphs represent the mean fold change in expression of NUPs upon infection relative to uninfected cells (lower panels A, B). **(C)** Differential infectivity of HIV-1 NL4.3. The left panel shows mean p55 expression in both SupT1 and HEK293T cells (from Figure 1C and Figure 2C, respectively) normalized to the loading control, GAPDH (from Figure 1A and Figure 2A, respectively). The right panel shows mean percentage of infectivity in HEK293T cells relative to SupT1 cells (100%). The experiments were performed at least three times. *, P<0.05; NS, P>0.05.


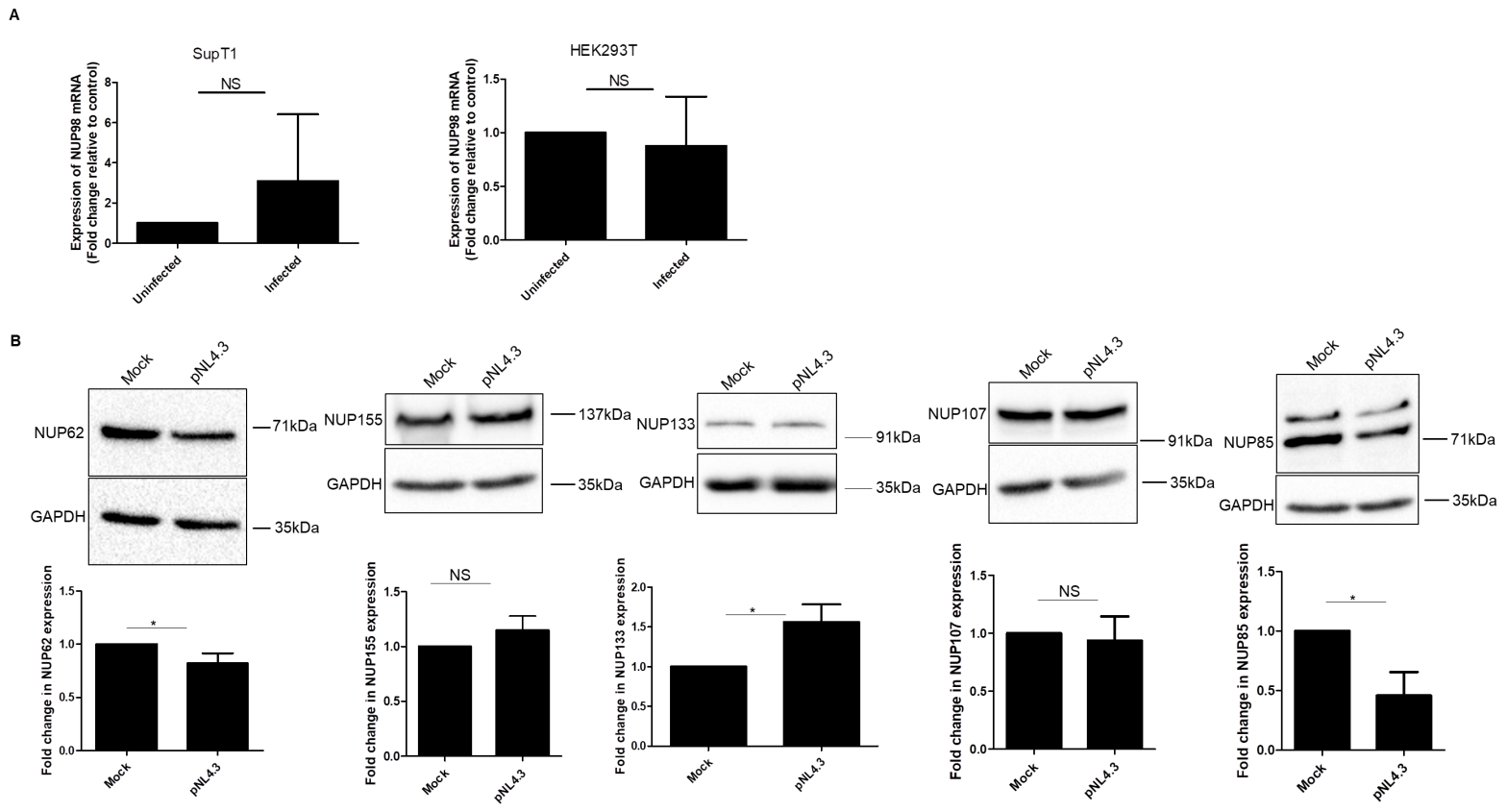


**Figure S2**. HIV-1 infection does not affect NUP98 mRNA in SupT1 and HEK293T cells. **(A)** SupT1 and HEK93T cells were infected with HIV-1 NL4.3 virus. Four days after infection, RNA was harvested. cDNA synthesized from the RNA was used as a template for the primers that amplify NUP98 and GAPDH reverse transcripts. The bar graphs represent the mean fold change in NUP98 mRNA relative to uninfected cells. **(B)** Differential expression of NUPs in HEK293T cells during transfection with pNL4.3. HEK293T cells were transfected with proviral plasmid pNL4.3. Two days after transfection, cells were lysed and the lysate was used for western blotting. Blots were probed with anti-NUP62, NUP155, NUP133, NUP107, NUP85 and anti-GAPDH antibodies (upper panel, B). The expression of the NUPs was normalized to the corresponding loading control, GAPDH. Bar graphs represent the mean fold change in expression of NUPs in pNL4.3 transfected cells relative to mock cells (lower panel, B). The experiments were performed at least three times. *, P<0.05; NS, P>0.05.


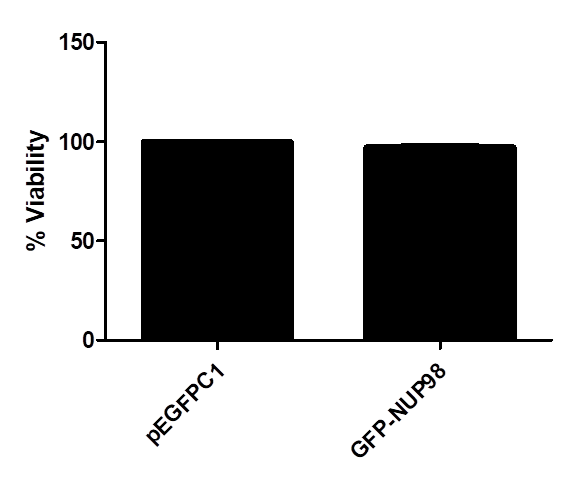


**Figure S3.** Overexpression of NUP98 does not affect cell viability. HEK293T cells were transfected with either pEGFPC1 or GFP-NUP98. Forty eight hours post-transfection, cells were harvested, stained with trypan blue and counted using hemocytometer. Bar graphs represent the percentage of viability of cells transfected with GFP-NUP98 relative to vector control (100%). The experiments were performed at least three times.


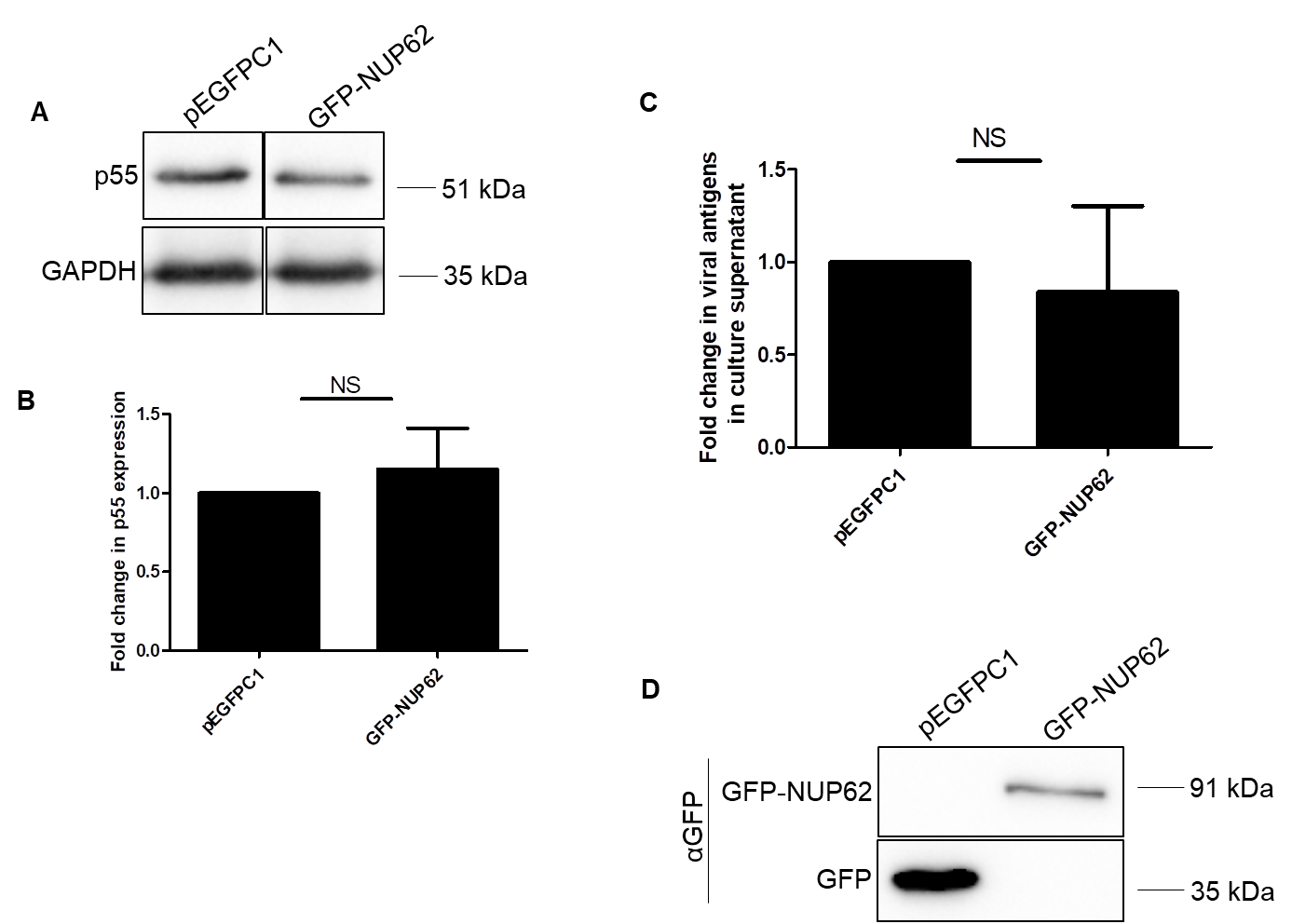


**Figure S4**. Overexpression of NUP62 does not affect viral protein levels and viral antigen release. **(A-D)** HEK293T cells were co-transfected with pNL4.3 and either pEGFPC1 or GFP-NUP62. Forty eight hours post transfection, the cells were harvested and culture supernatant was collected. Cells were lysed and the lysates were used for western blotting. **(A)** Blots were probed with anti-HIV-1 p24 and GAPDH antibodies. **(B)** p55 expression was normalized to the loading control, GAPDH and the bar graphs represent the mean fold change in expression of p55 relative to the vector control. **(C)** The viral antigens from the culture supernatant was estimated by p24 ELISA and the bar graphs represent the mean fold change of viral antigens relative to the vector control. **(D)** The GFP-NUP62 expression was verified by western blotting using anti-GFP antibody. The experiments were performed at least three times. NS, P>0.05.


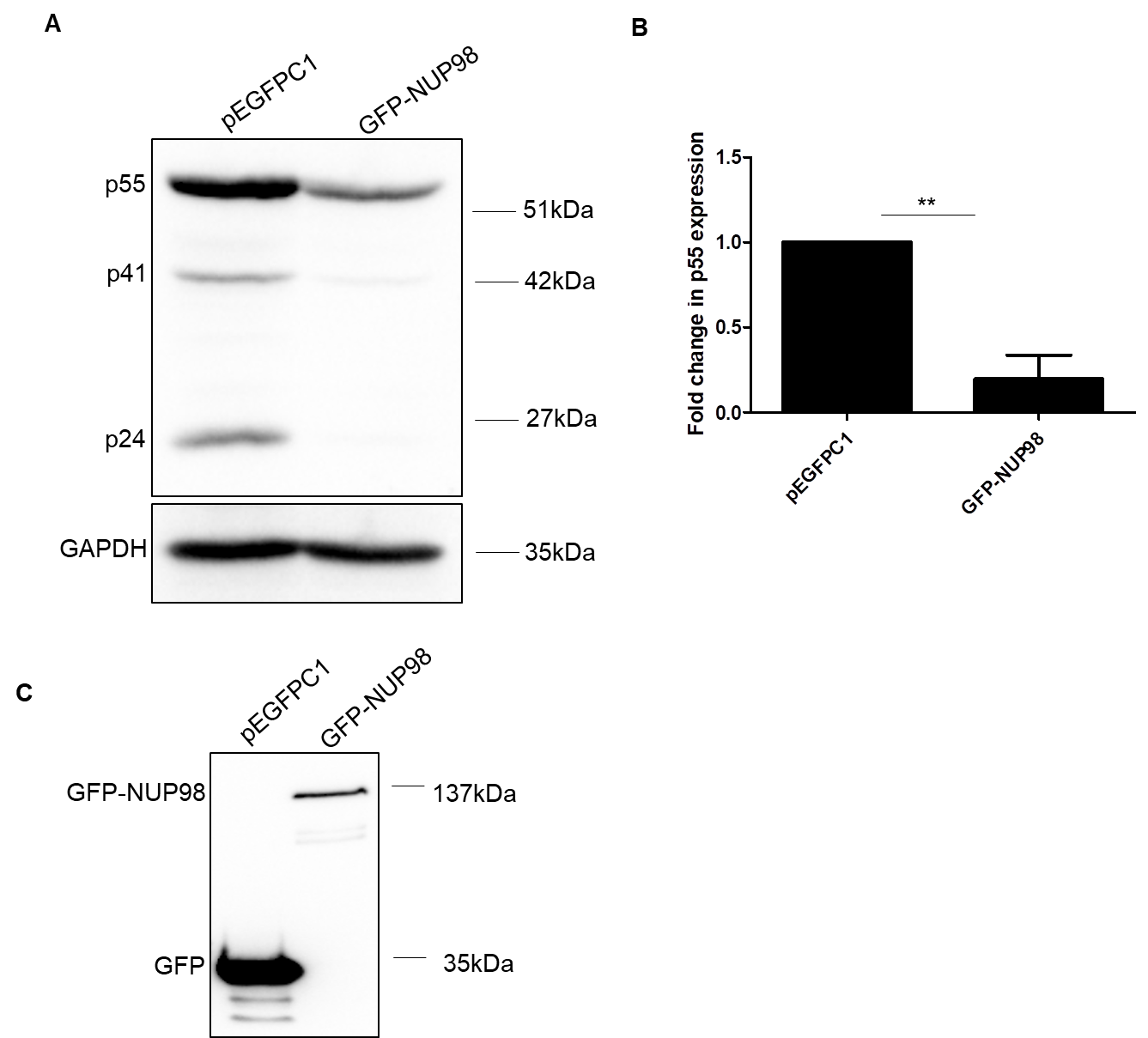


**Figure S5**. NUP98 negatively affects HIV-1 subtype C (Indie-C1) viral protein levels in HEK293T cells. **(A-C)** HEK293T cells were co-transfected with proviral plasmid pIndie-C1 and pEGFPC1 or GFP-NUP98. Two days after transfection, the cells were lysed and the lysates were used for western blotting. **(A)** Blots were probed with anti-HIV-1 p24 and anti-GAPDH antibodies. **(B)** p55 expression was normalized to the loading control GAPDH and the bar graphs represent the mean fold change in the expression of p55 relative to the pEGFPC1 transfected cells. **(C)** The expression of GFP constructs was verified by western blotting using anti-GFP antibody; **, P<0.01.


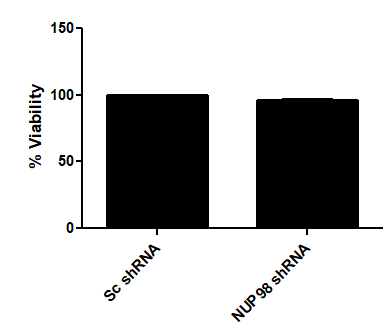


**Figure S6**. Cell viability in NUP98 depleted SupT1 cells. Seventy two hours post-transduction of SupT1 with lentivirus containing either Sc shRNA or NUP98 shRNA, cells were harvested, stained with trypan blue and counted using haemocytometer. The bar graphs represent the mean percentage viability of cells transduced with NUP98 shRNA relative to Sc shRNA control (100%).


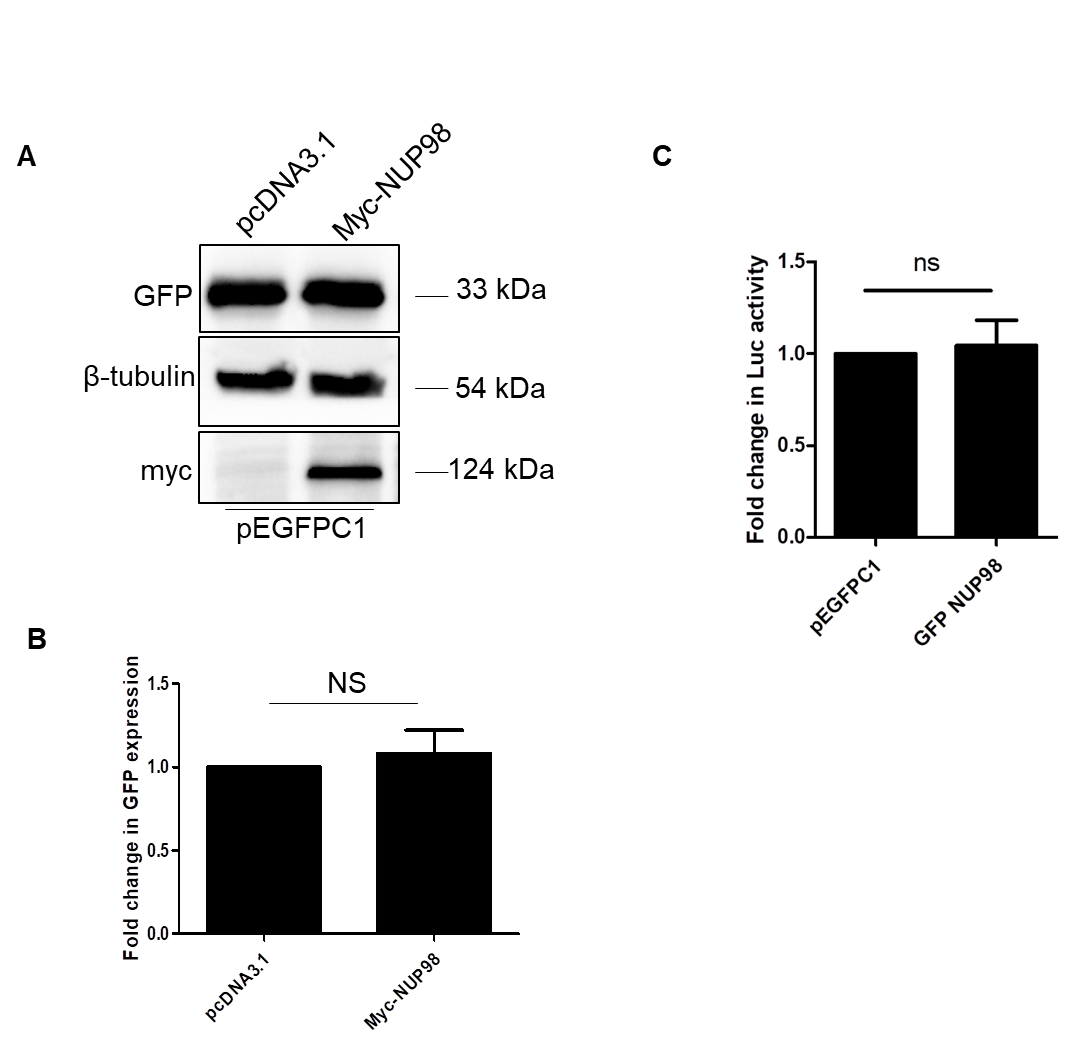


**Figure S7**. NUP98 does not influence CMV and SIV promoter activities. **(A, B)** HEK293T cells were co-transfected with pEGFPC1 and either pcDNA or Myc-NUP98. Two days after transfection, the cells were lysed and lysates were used for western blotting. **(A)** Blots were probed with anti-GFP, anti-β-tubulin and anti-Myc antibodies. **(B)** GFP expression was normalized to the loading control β-tubulin and the bar graphs represent the mean fold change in expression of GFP relative to the vector control. **(C)** HEK293T cells were co-transfected with pEGFPC1 or GFP NUP98 and pSIV_AGM_-Luc-R^−^E^−^Δvif. Two days after transfection, the cells were lysed and the lysate was used for luciferase activity. The bar graphs represent the mean fold change in luciferase activity relative to vector control. The experiments were performed at least three times. NS, P>0.05.


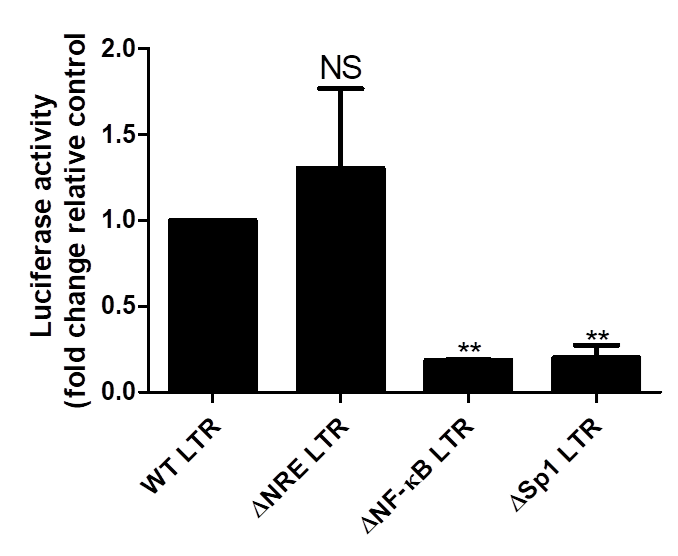


**Figure S8**. Basal promoter activity of HIV-1 LTR mutants. HEK293T cells were co-transfected with pEGFPC1 and pLTR-Luc or deletion mutant LTR plasmid constructs. Forty eight hours post transfection, the cells were lysed in reporter lysis buffer and the lysate was used for luciferase activity. The bar graphs represent the mean basal luciferase activities of LTR mutant constructs relative to wild type (WT) LTR vector. The experiments were performed at least three times. **, P<0.01; NS, P>0.05.


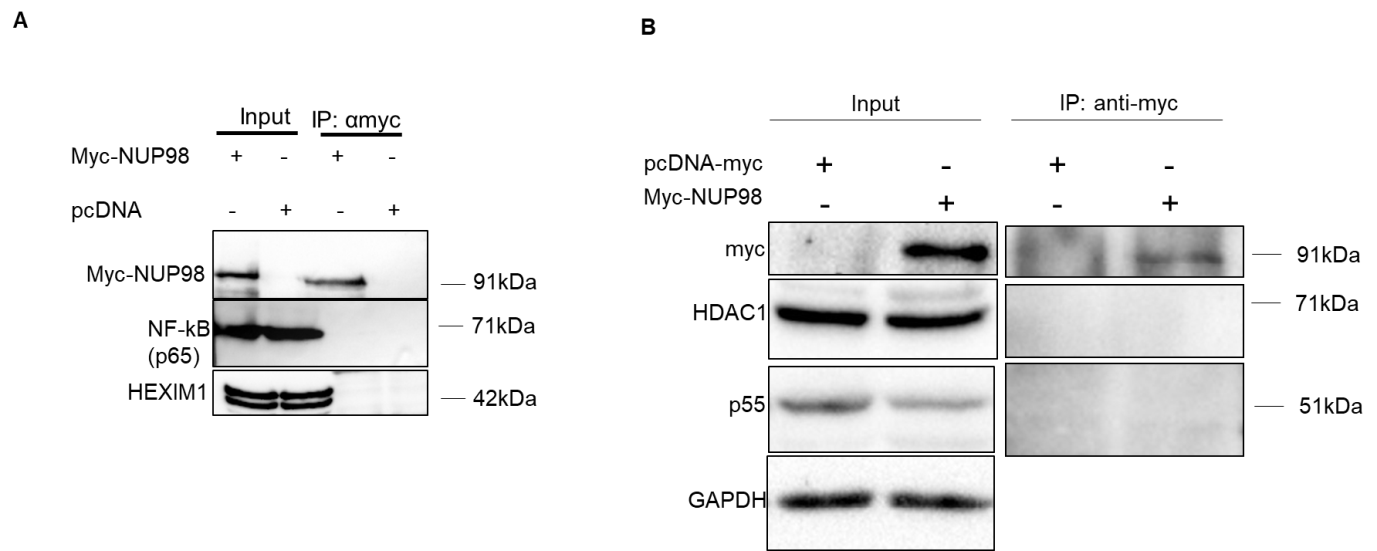


**Figure S9**. NUP98 does not interact with NF-κB subunit p65, HEXIM1 or HDAC1. **(A)** HEK293T cells were co-transfected with pNL4.3 and either pcDNA or Myc-NUP98. Two days after transfection, the cells were lysed and the lysate was used for co-immunoprecipitation by anti-myc antibody and followed by western blotting. The presence of Myc-NUP98, p65 and HEXIM1 in the immunoprecipitates was assessed by probing the blots with anti-Myc, anti-p65 and anti-HEXIM1 antibodies. **(B)** HEK293T cells infected with HIV-1 NL4.3 were transfected with either pcDNA or Myc-NUP98. Two days after transfection, the cells were lysed and the lysate was used for co-immunoprecipitation by anti-Myc antibody and followed by western blotting. The presence of Myc-NUP98, HDAC1 and p55 in the immunoprecipitates was assessed by probing the blots with anti-Myc, anti-HDAC1 and anti-HIV-1 p24 antibodies, respectively.


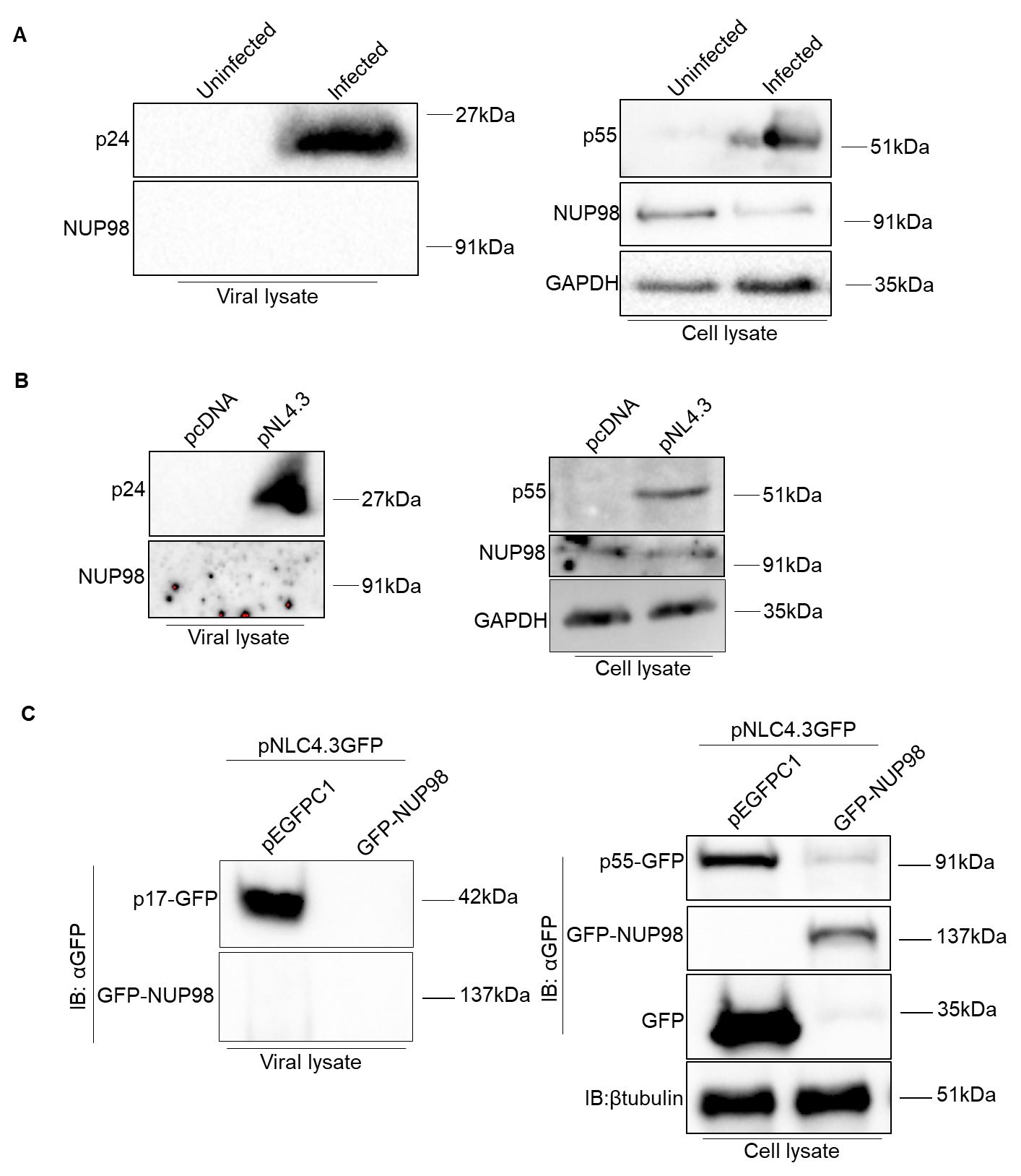


**Figure S10**. NUP98 is not co-packaged into the released viral particles. **(A)** SupT1 cells were infected with HIV-1 NL4.3, and four days post-infection, cells were harvested and the culture supernatant was collected. Culture supernatant containing the viral particles was subjected to PEG precipitation as described in methods and resuspended in NP40 lysis buffer. The resulting viral lysates and cell lysates were probed with antibodies against HIV-1 p24, GAPDH and NUP98. **(B)** HEK293T cells were transfected with either pcDNA or pNL4.3. Forty eight hours post-transfection, cells were harvested and the culture supernatant was collected. Culture supernatant containing the viral particles was subjected to PEG precipitation and the resulting viral pellet was resuspended in NP40 lysis buffer. The viral and cell lysates were probed with anti-HIV-1 p24, anti-GAPDH and anti-NUP98 antibodies. **(C)** HEK293T cells were co-transfected with pNLC4.3GFP and either pEGFPC1 or GFP-NUP98 and processed as described in (**B)**. The viral and the cell lysates were probed with anti-GFP and anti-tubulin antibodies.
